# Supplementary material for: Hybrid metagenome assemblies link carbohydrate structure with function in the human gut microbiome
Source: Commun Biol. 2022 Sep 8;5:932. doi: 10.1038/s42003-022-03865-0 (PMC9458734; doi:10.1038/s42003-022-03865-0)
Supplement: Supplementary file 2 — Supplementary Information [file 42003_2022_3865_MOESM2_ESM.pdf]

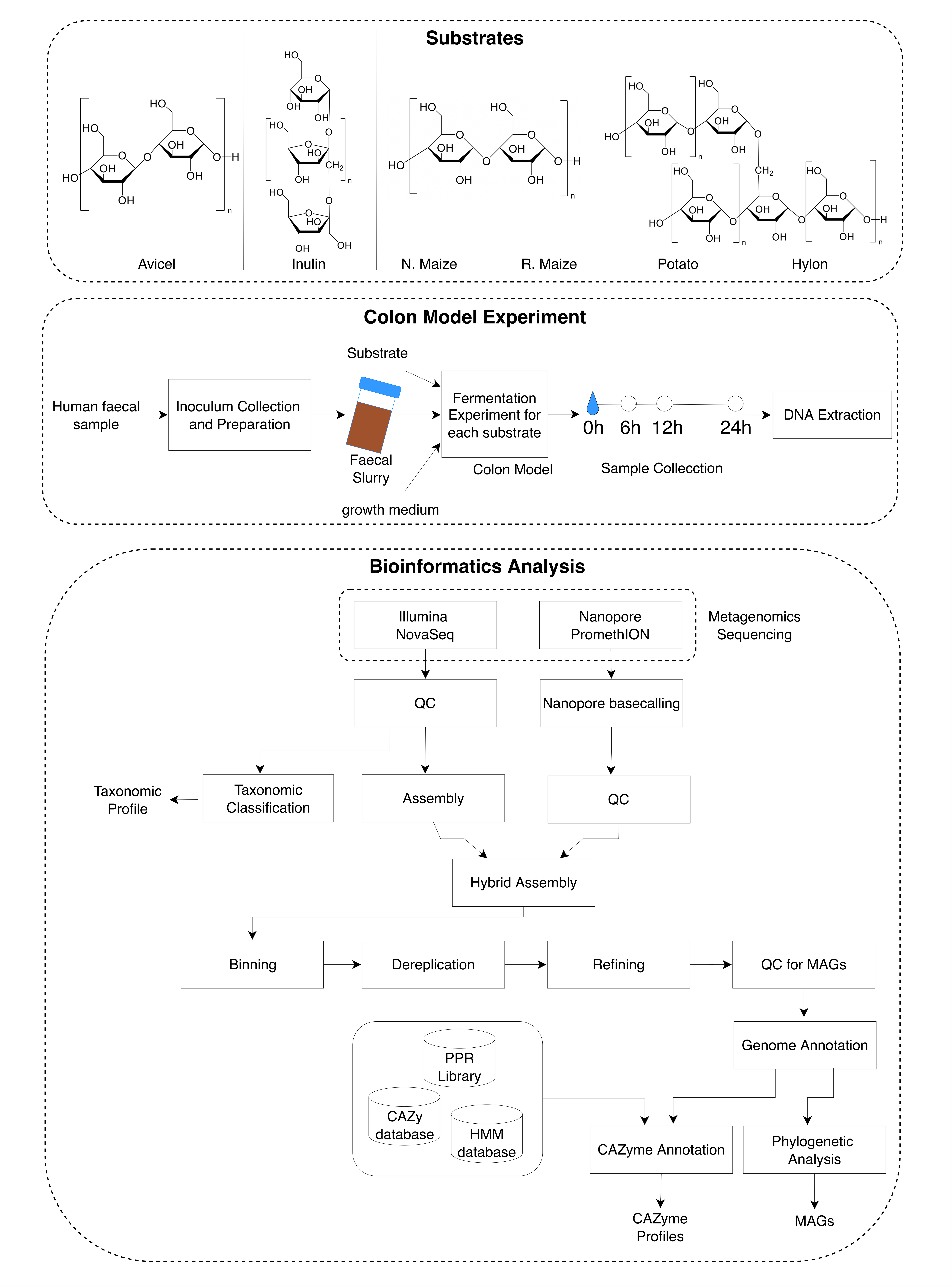

Supplementary Figure 1: Extended detailed workflow for bioinformatics analysis of combined Illumina NovaSeq and Oxford Nanopore PromethION metagenomics data collected in a model colon study of the fermentation of different carbohydrate substrates with contrasting structures (Avicel, Inulin, Normal maize (N.maize), Retrograded maize (R.maize), Potato and Hylon) by the gut microbiota present in a human stool sample.

Supplementary figure 2

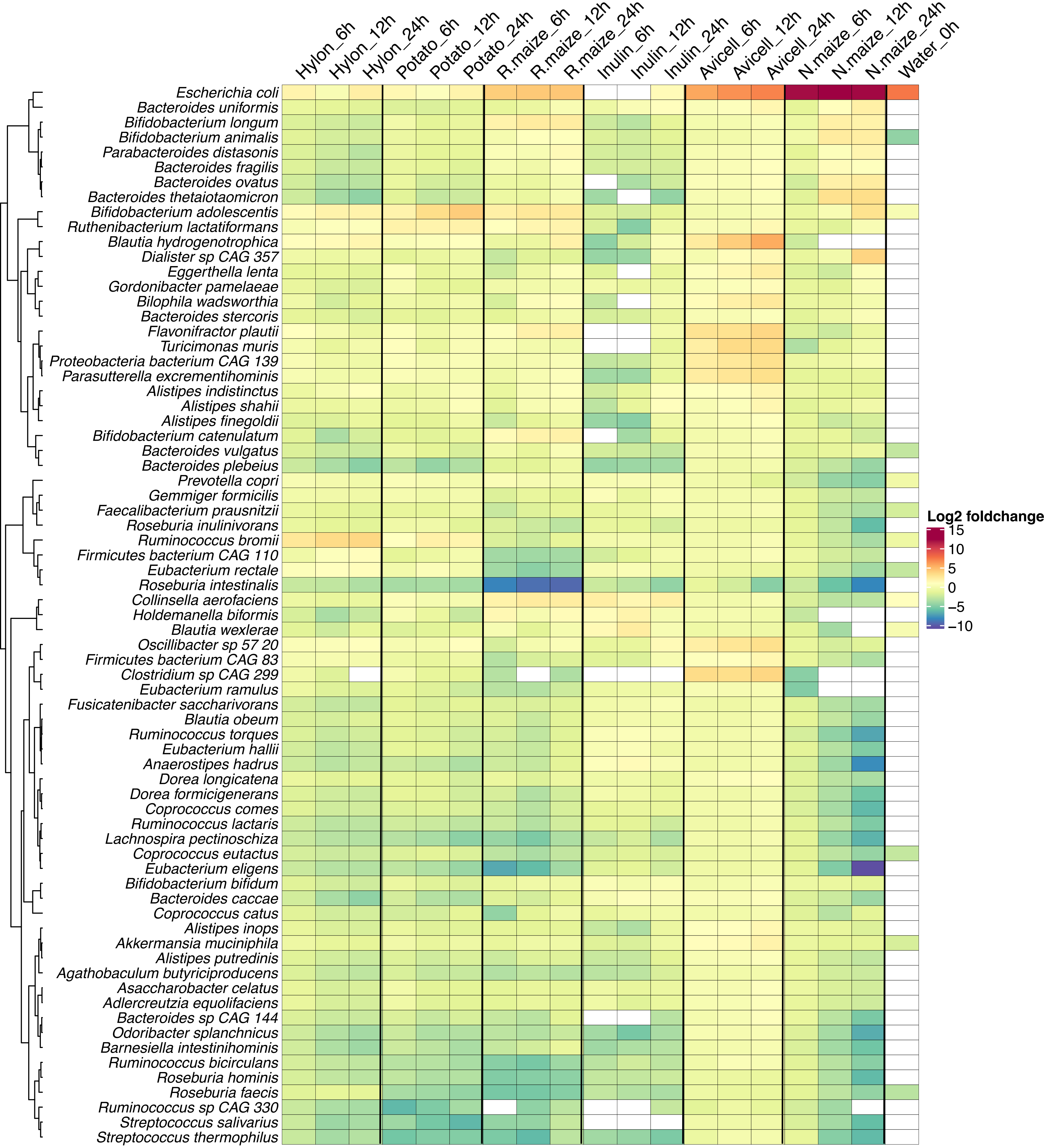

Supplementary Figure 2: Hierarchical clustering of the log2 fold-change of gut microbial species present after fermentation of Avicel, Inulin, N.maize, R.maize, Potato and Hylon at 0h, 6h, 12h and 24h in the model colon. The hierarchical clustering also includes a water sample (the kitome).

Supplementary figure 3

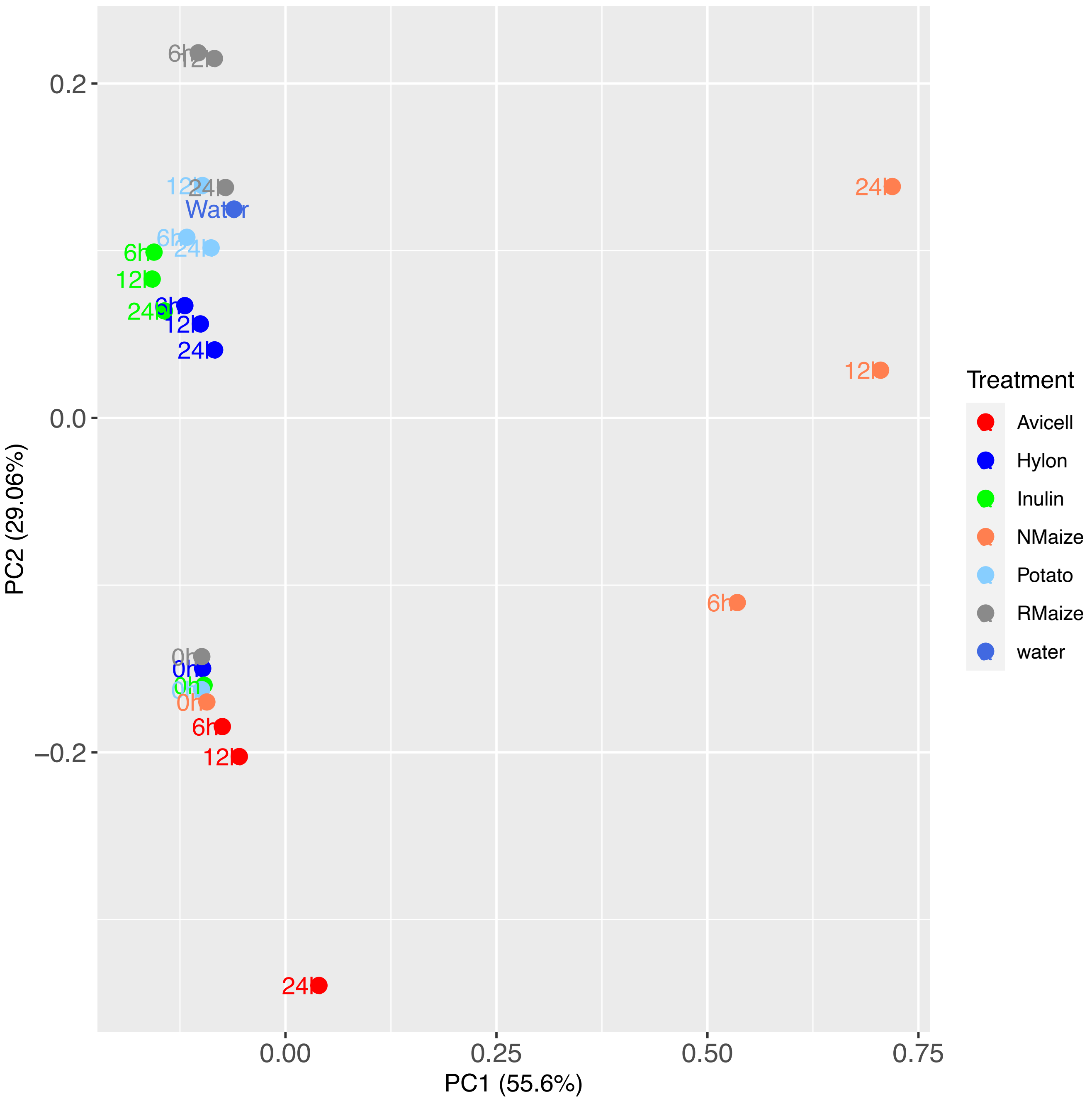

Supplementary Figure 3: Principle Component Analysis (PCoA) of all treatments showing the dynamics of the microbiome during the different time points and between the Carbohydrate treatment. PC1 and PC2 represent the percentage of variance explained by Principle Component (PC) 1 and 2.

Supplementary figure 4

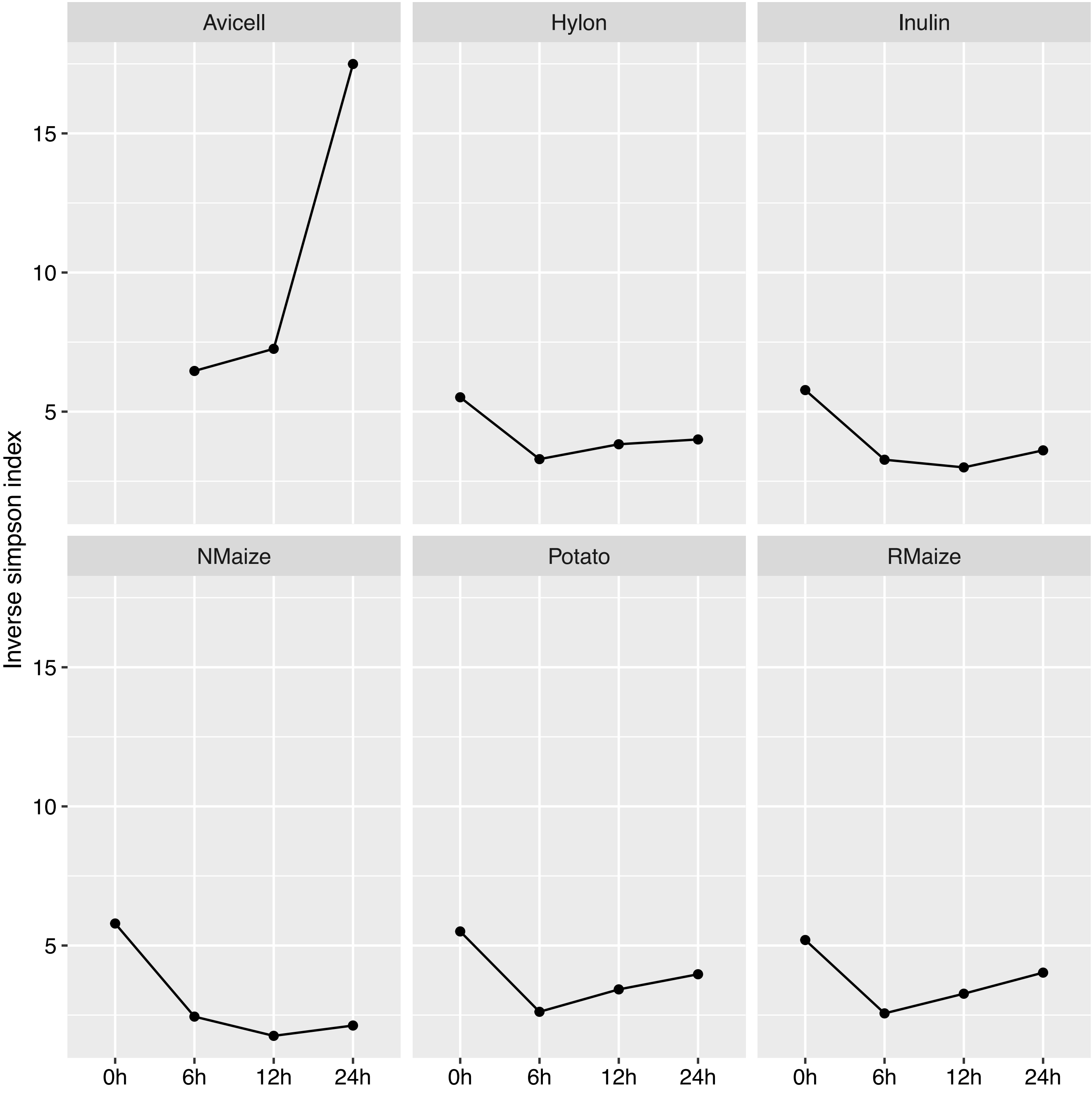

Supplementary Figure 4: Changes in inverse Simpson index between time periods of the substrates.

Supplementary figure 5

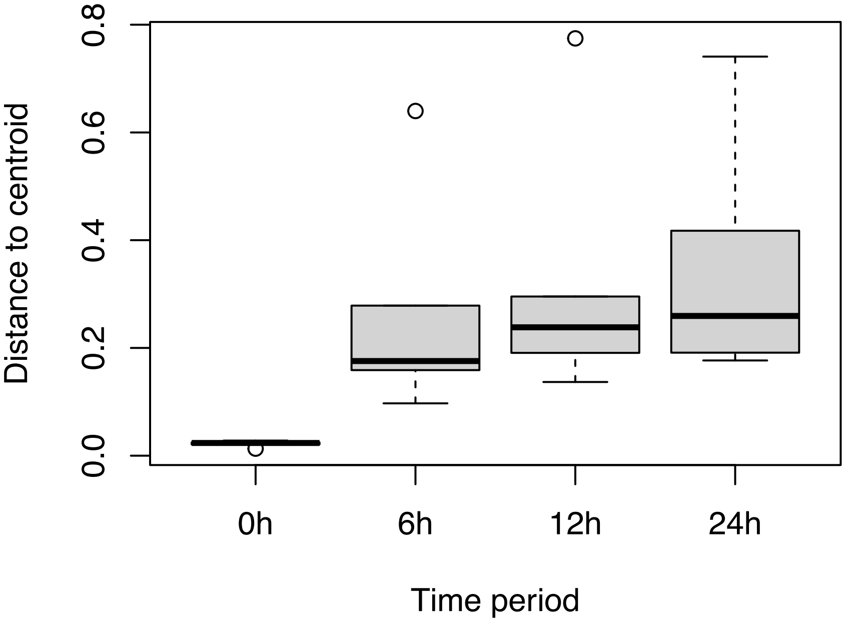

Supplementary Figure 5: Empirical distances between microbial communities. The boxplot with whiskers represents the distances calculated between microbial communities at each timepoint, relative to the centroid. The box represents the interquartile range (IQR) (25th and 75th percentile); the median is shown within the box. The whiskers indicate minimum and maximum Inter Quartile Range (IQR); dots represent outliers.

Supplementary figure 6

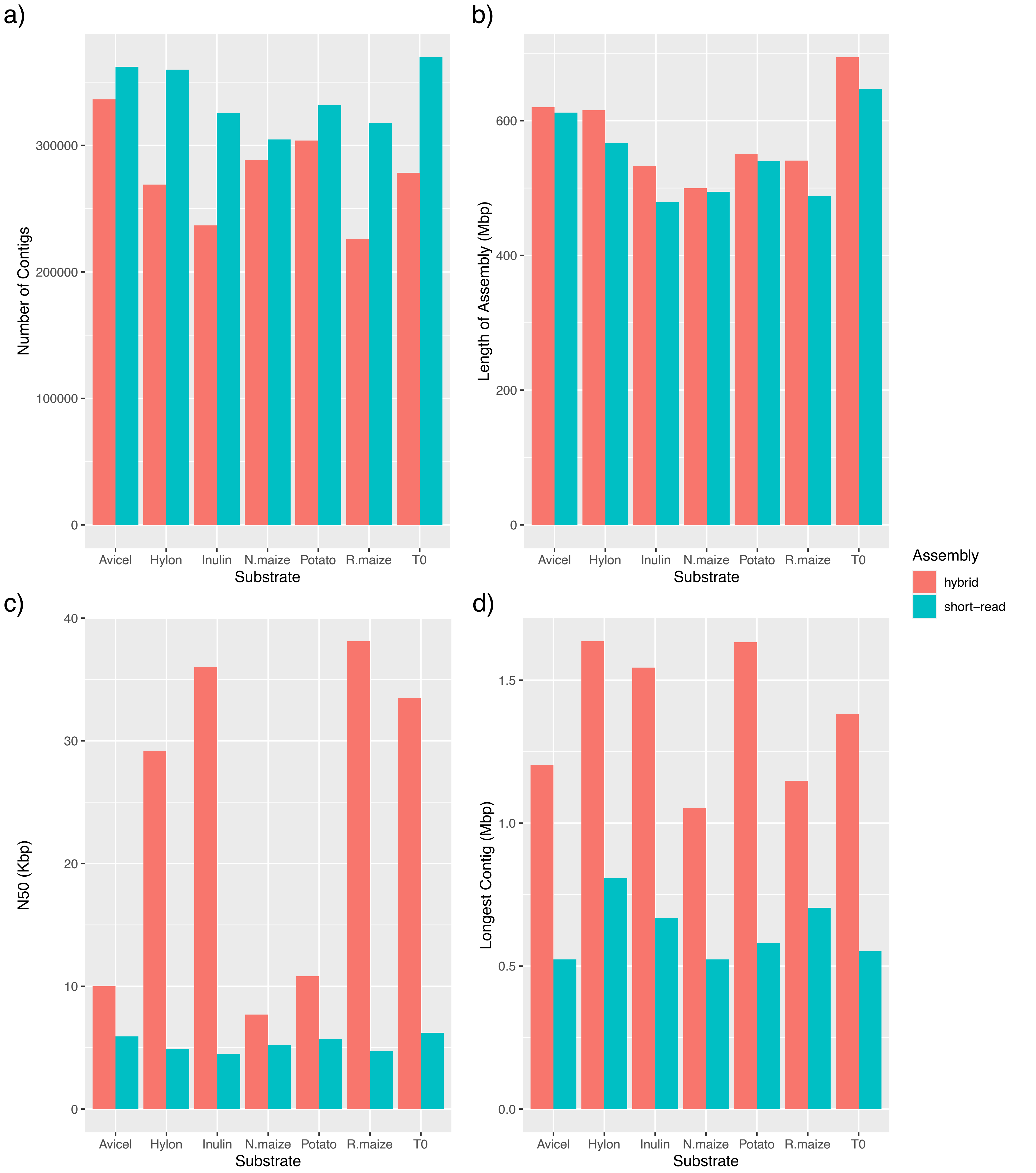

Supplementary Figure 6: Comparison of Illumina short read assemblies and hybrid assemblies: a) shows the number of contigs per treatment, b) shows the N50, c) statistics on the largest contig, d) size of the total assembly for each carbohydrate treatment.

Supplementary figure 7

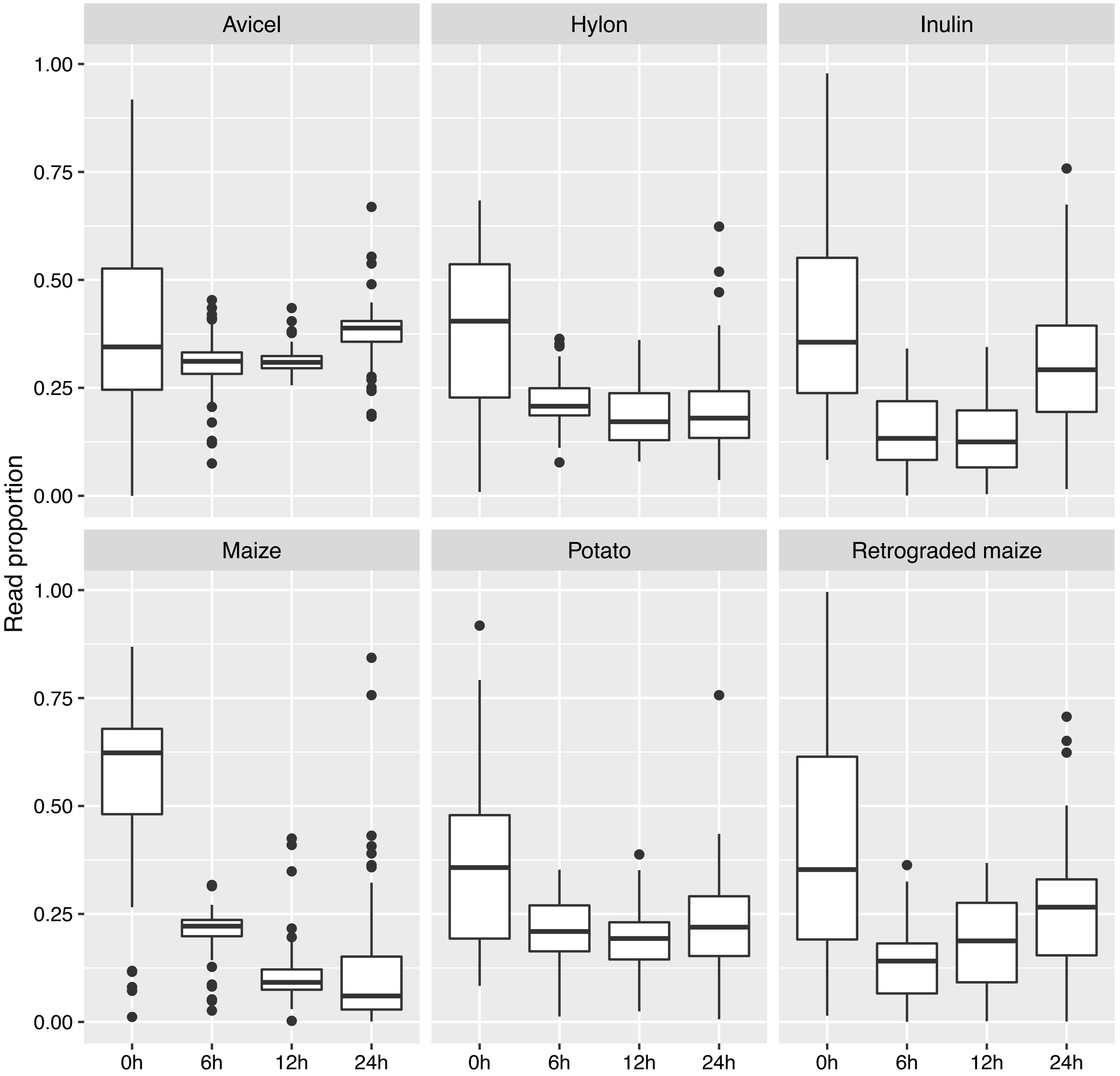

Supplementary Figure 7: Box plots showing the dynamic shifts in read proportions for all binned MAGs after 0h, 6h, 12h and 24h fermentation in the model colon. The box represents the interquartile range (IQR) (25th and 75th percentile); the median is shown within the box. The whiskers indicate minimum and maximum Inter Quartile Range (IQR); dots represent outliers.

Supplementary figure 8

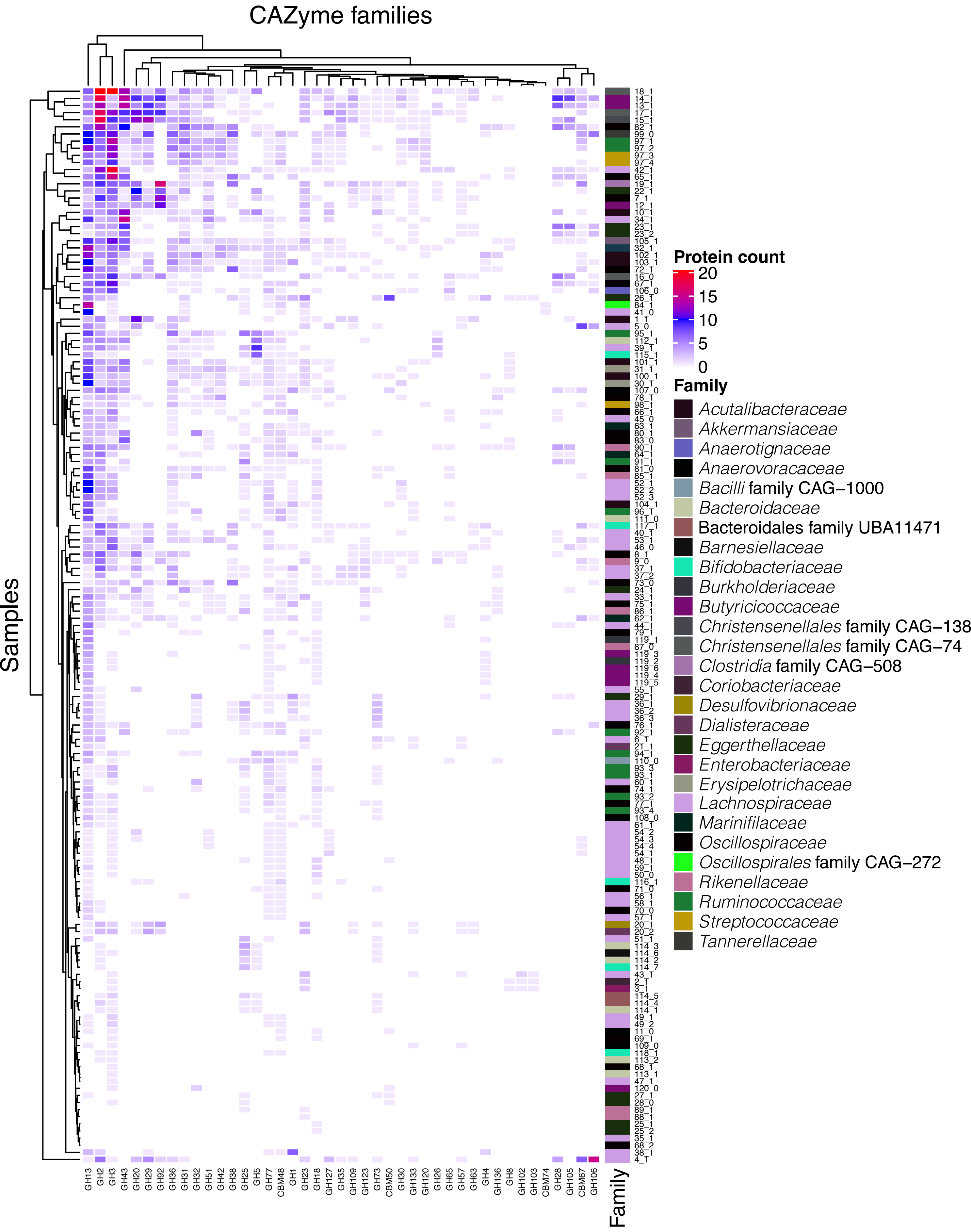

Supplementary Figure 8: Distribution of CAZy families per substrate and in all the genomes.

Supplementary figure 9

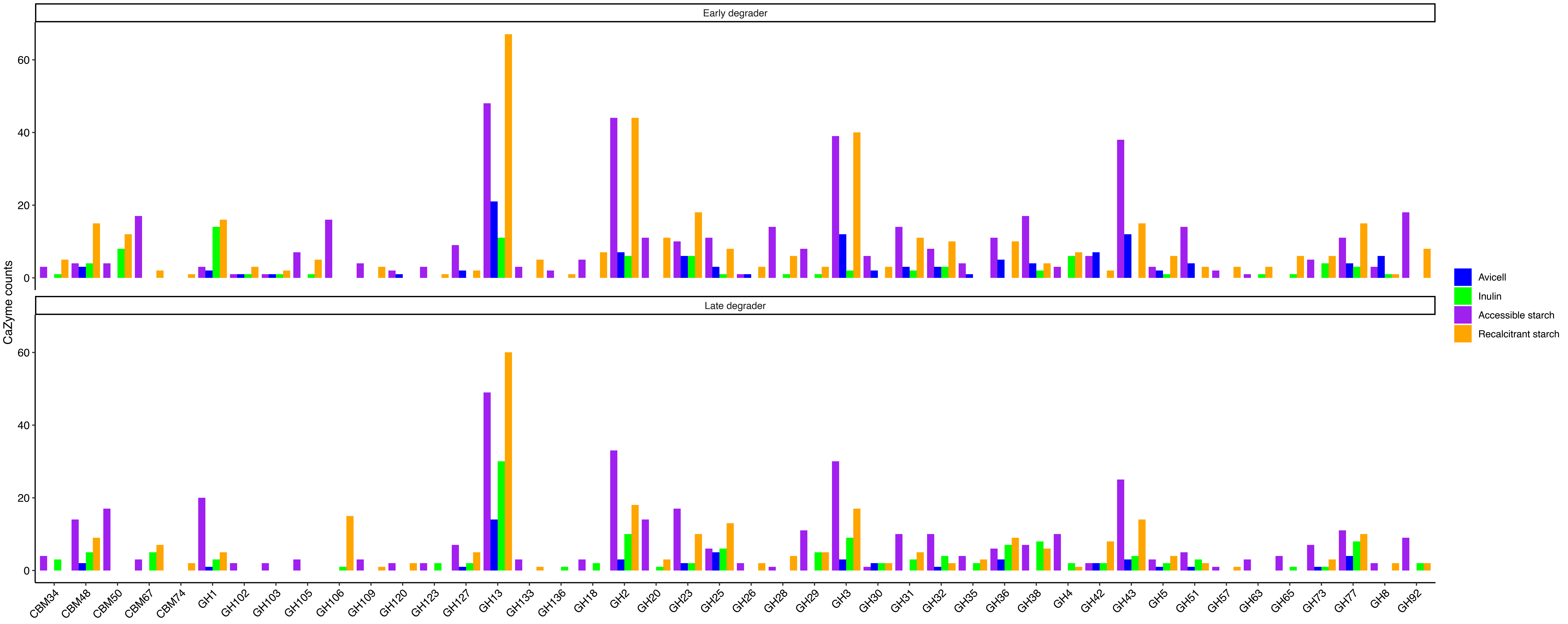

Supplementary Figure 9: Combined CAZyme counts in MAG's for early and late degraders

Supplementary Figure 10

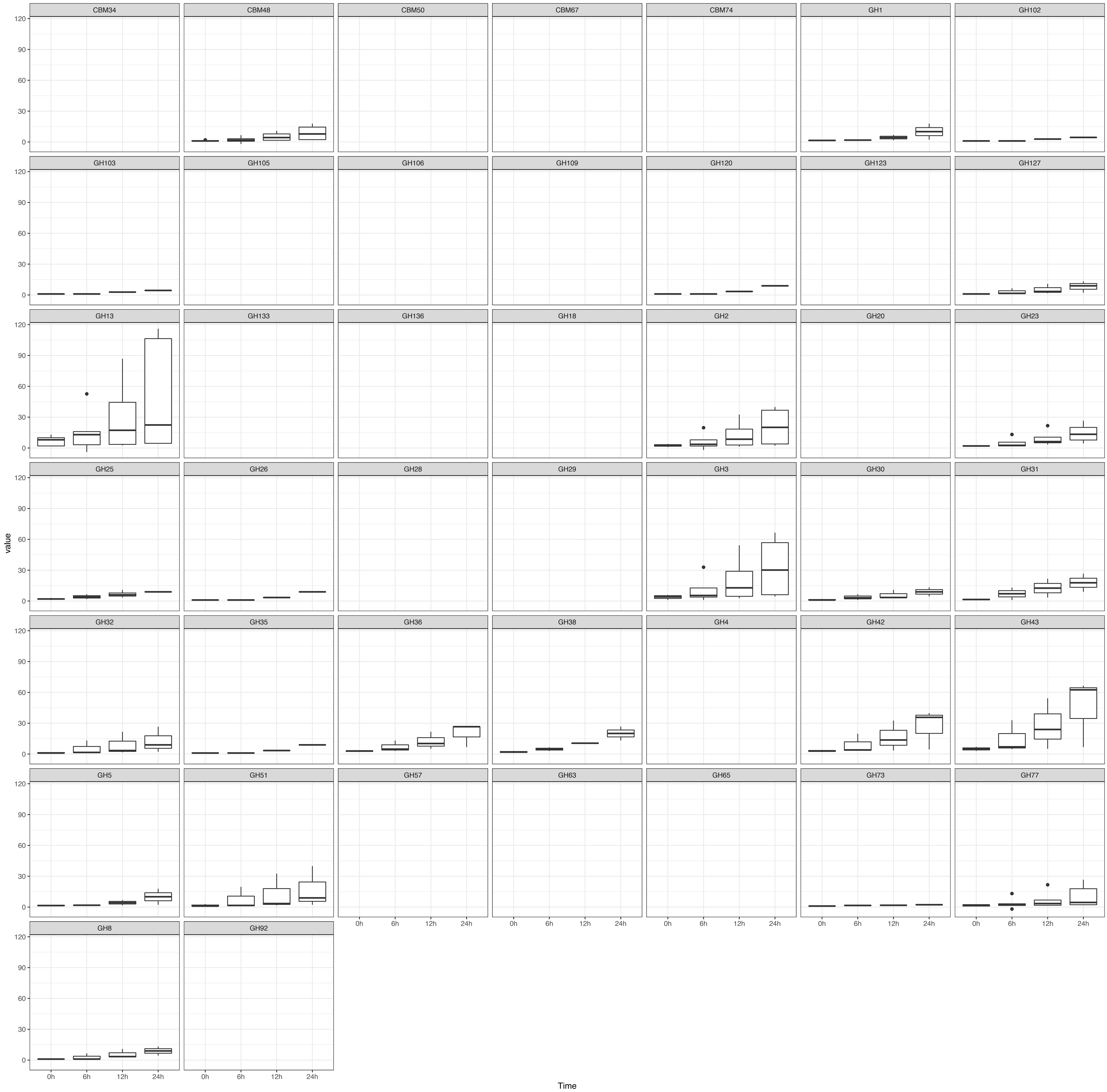

Supplementary figure 10: Box plots indicating the abundance of CaZymes relative to the abundance of each MAG in Avicell

Supplementary figure 11

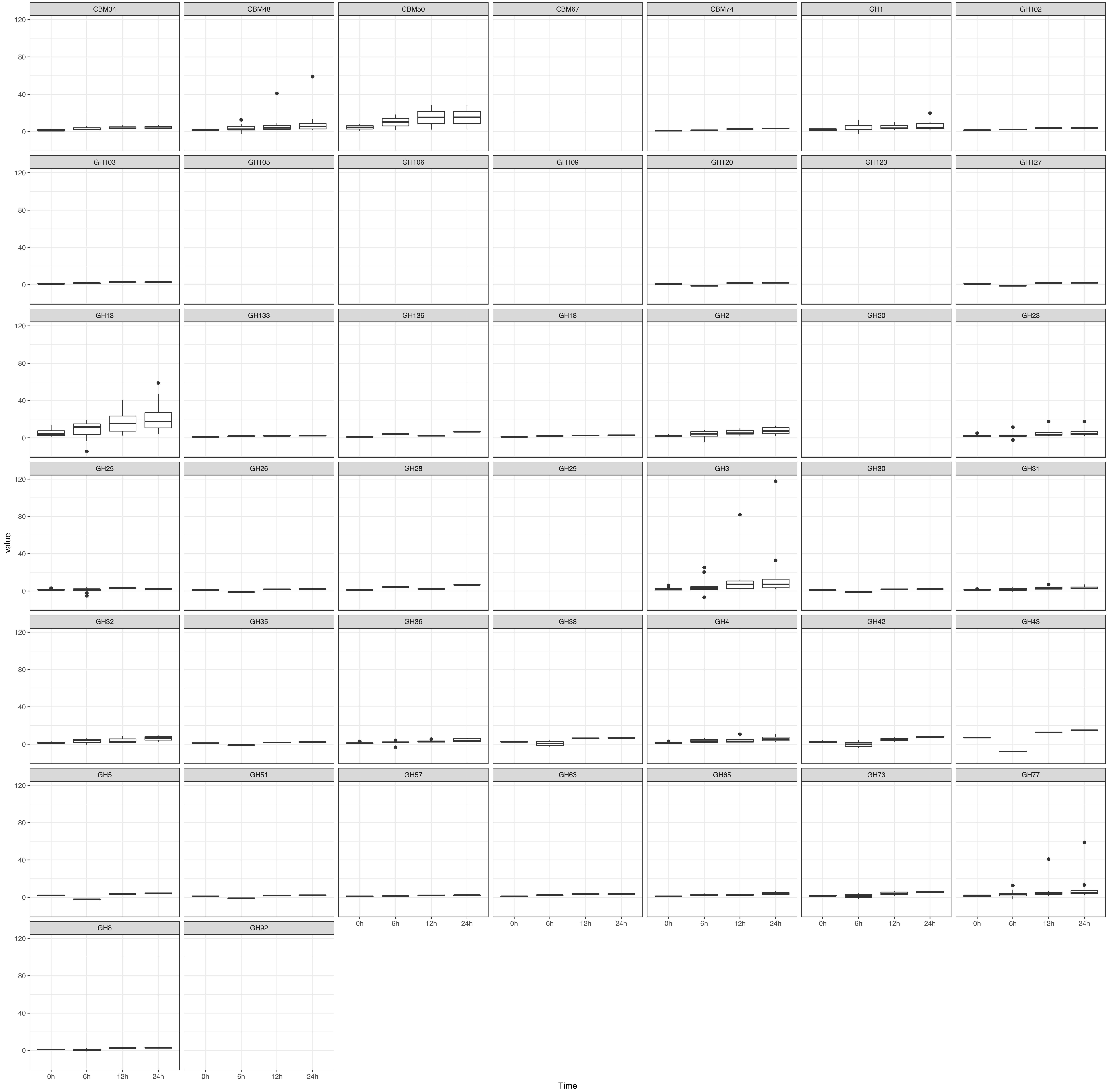

Supplementary figure 11: Box plots indicating the abundance of CaZymes relative to the abundance of each MAG in hylon. The box represents the interquartile range (IQR) (25th and 75th percentile); the median is shown within the box. The whiskers indicate minimum and maximum Inter Quartile Range (IQR); dots represent outliers.

Supplementary figure 12

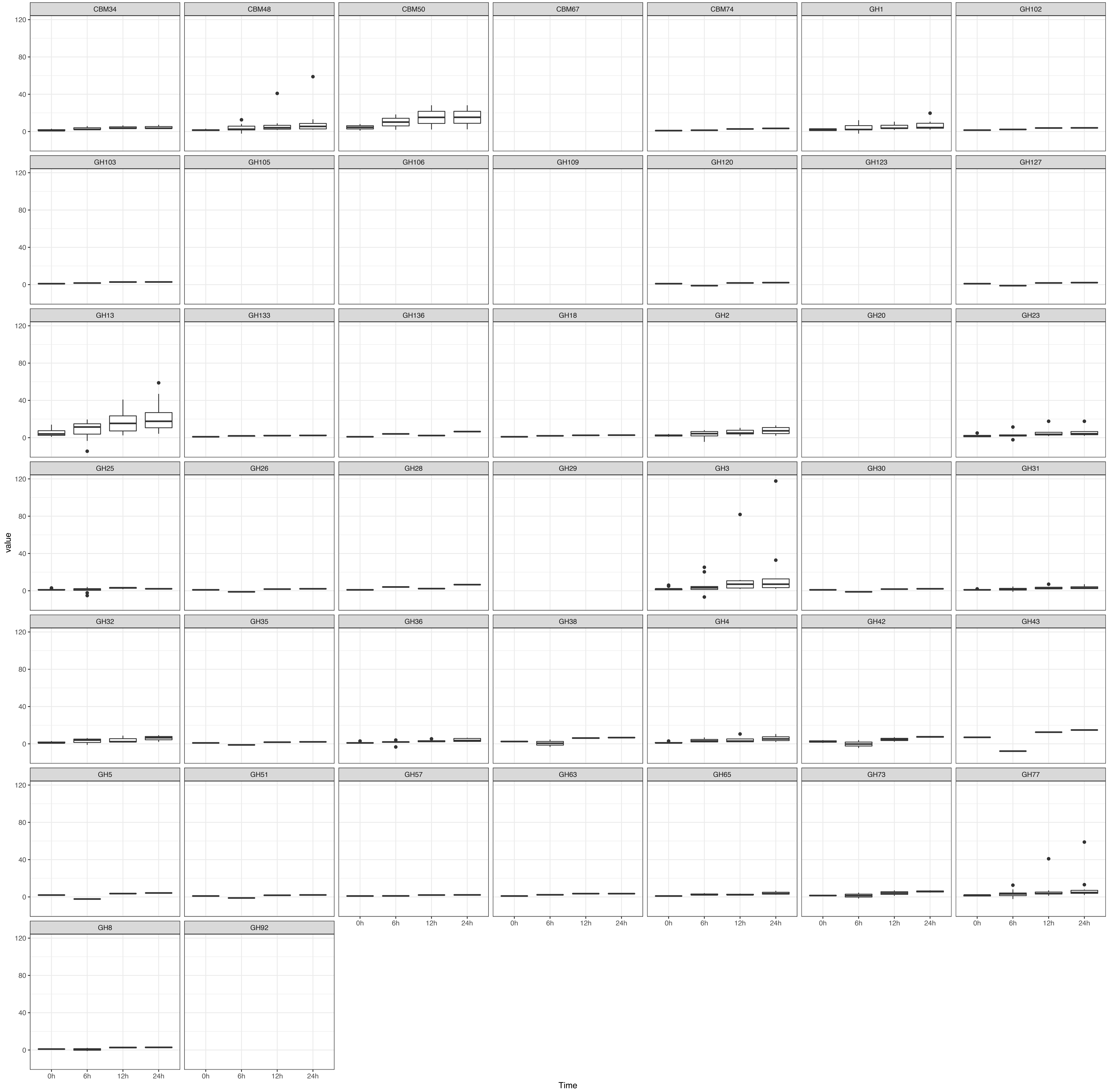

Supplementary figure 12: Box plots indicating the abundance of CaZymes relative to the abundance of each MAG in inulin. The box represents the interquartile range (IQR) (25th and 75th percentile); the median is shown within the box. The whiskers indicate minimum and maximum Inter Quartile Range (IQR); dots represent outliers.

Supplementary figure 13

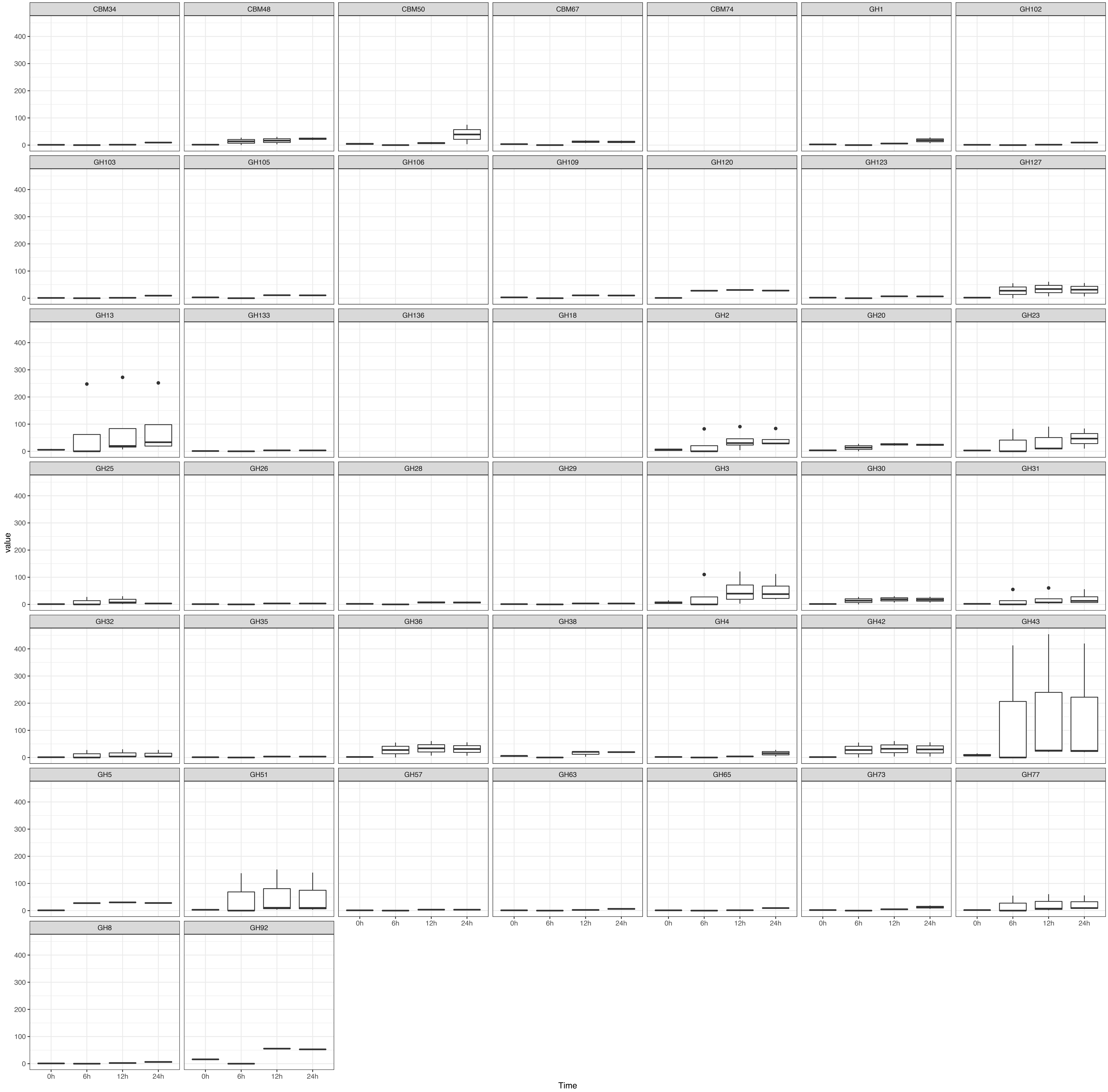

Supplementary figure 13: Box plots indicating the abundance of CaZymes relative to the abundance of each MAG in normal maize. The box represents the interquartile range (IQR) (25th and 75th percentile); the median is shown within the box. The whiskers indicate minimum and maximum Inter Quartile Range (IQR); dots represent outliers.

Supplementary figure 14

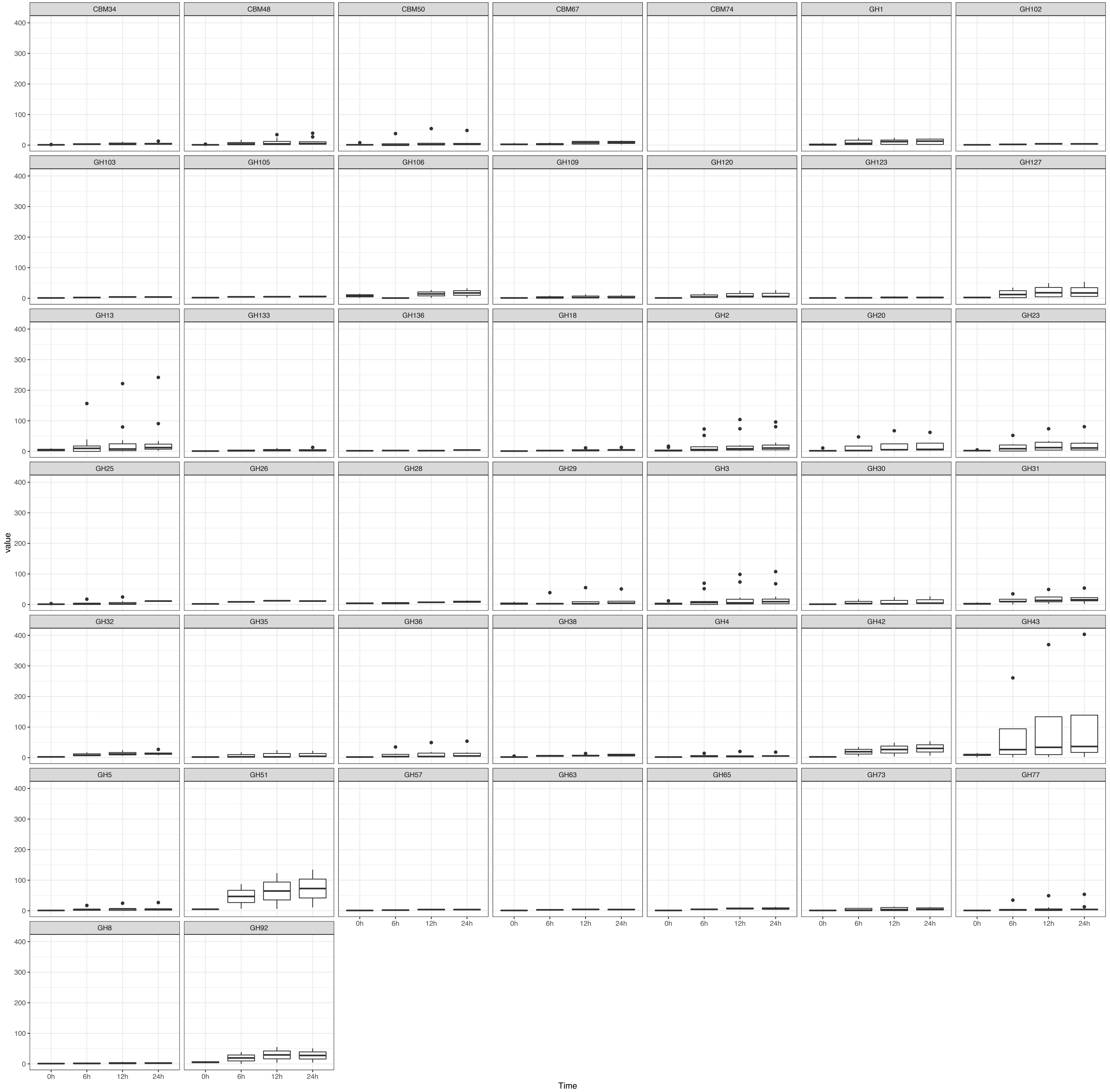

Supplementary figure 14: Box plots indicating the abundance of CaZymes relative to the abundance of each MAG in retrograded maize. The box represents the interquartile range (IQR) (25th and 75th percentile); the median is shown within the box. The whiskers indicate minimum and maximum Inter Quartile Range (IQR); dots represent outliers.

Supplementary figure 15

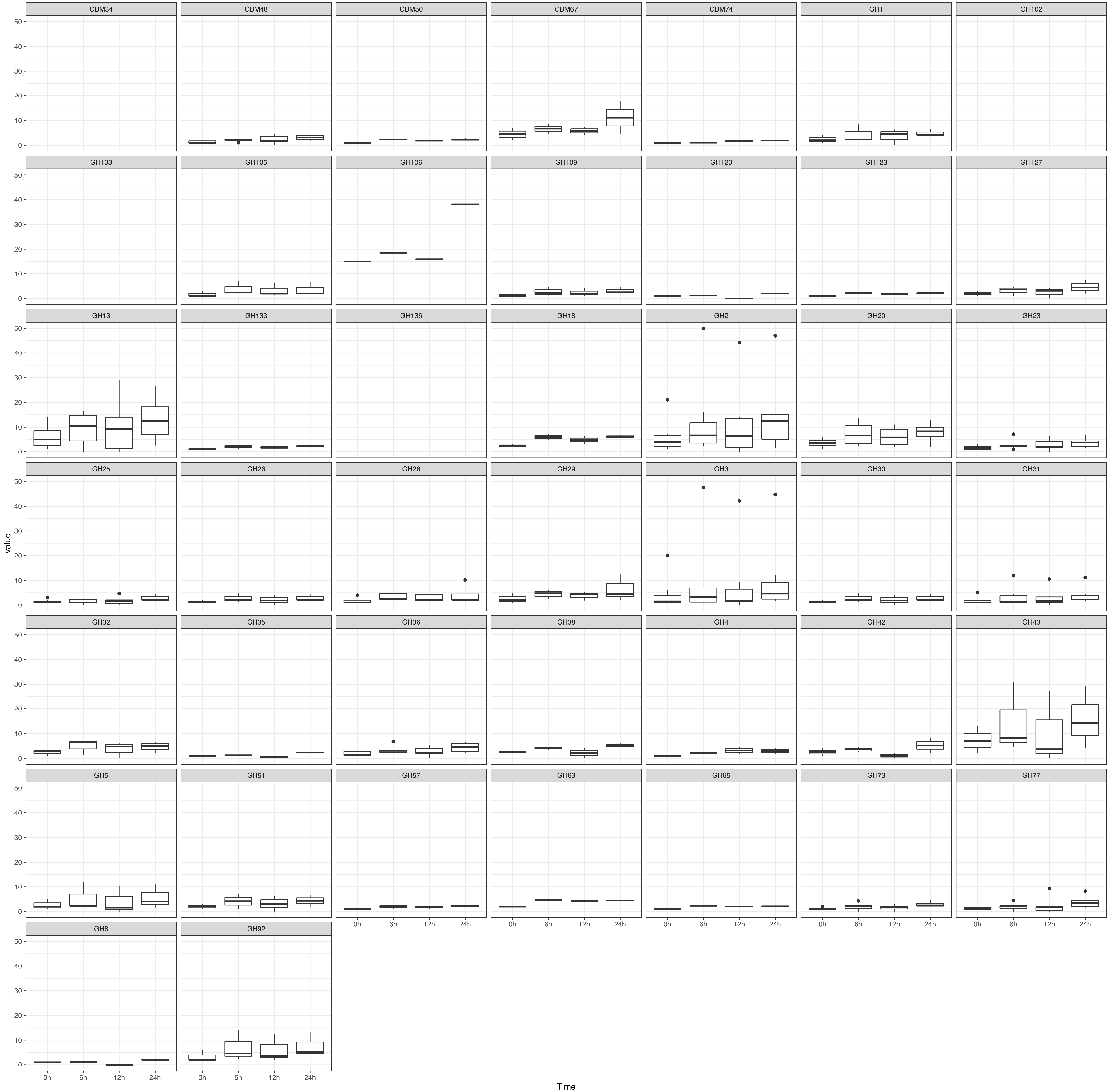

Supplementary figure 15: Box plots indicating the abundance of CaZymes relative to the abundance of each MAG in potato. The box represents the interquartile range (IQR) (25th and 75th percentile); the median is shown within the box. The whiskers indicate minimum and maximum Inter Quartile Range (IQR); dots represent outliers.

Supplementary figure 16

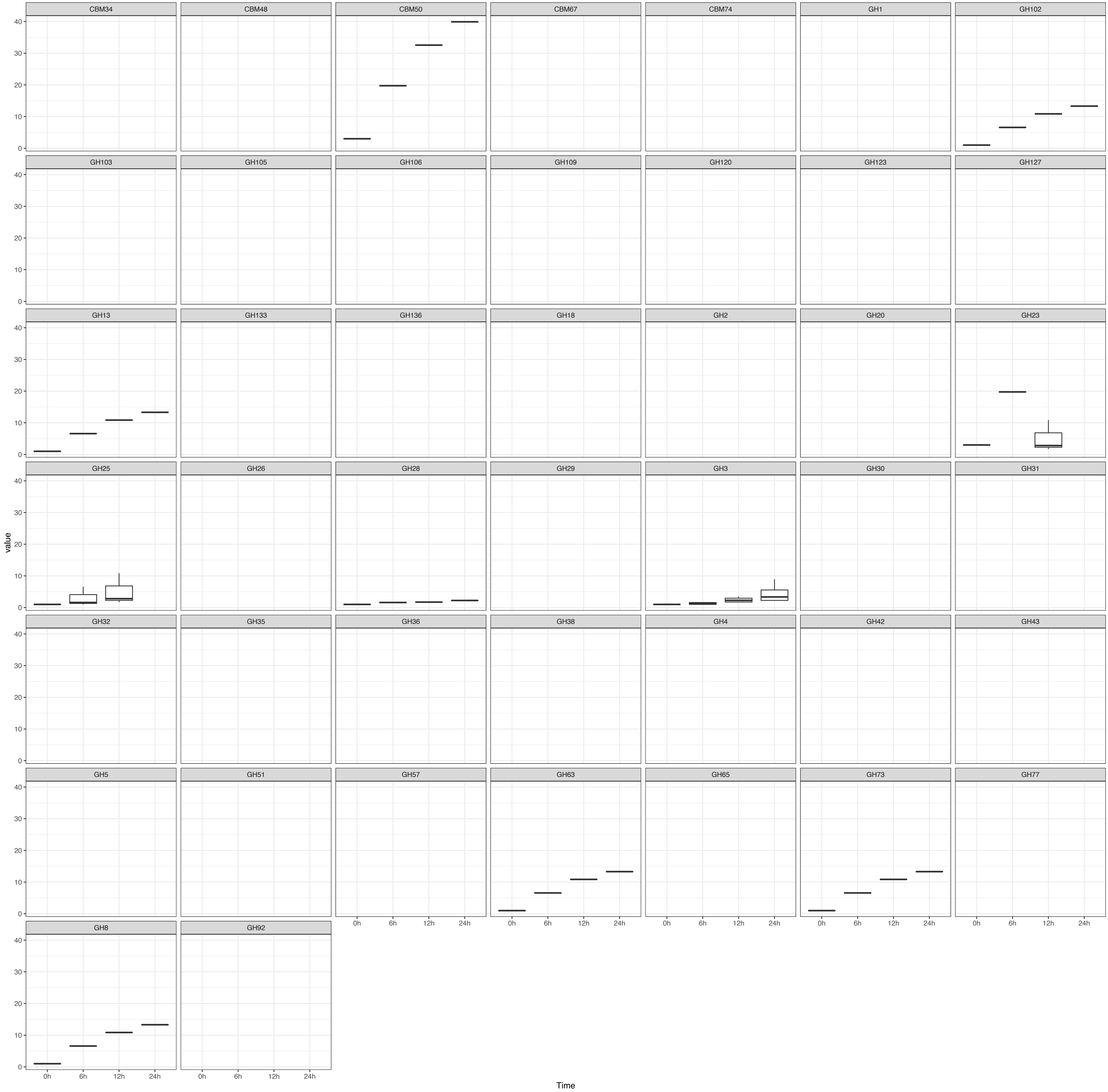

Supplementary figure 16: Patterns indicating the abundance of CaZymes that have a Signal peptide relative to the abundance of each MAG in Avicell

Supplementary figure 17

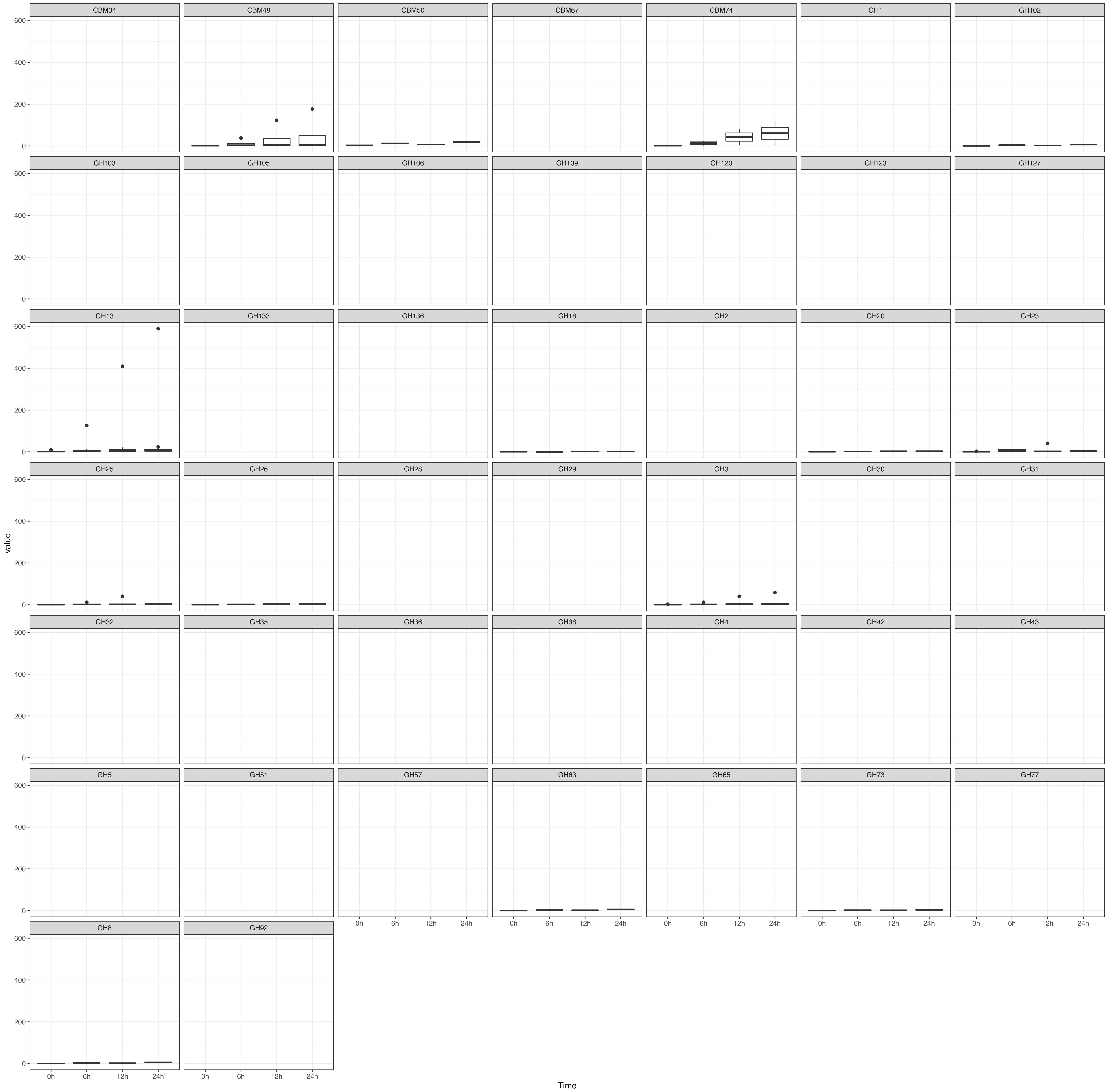

Supplementary figure 17: Patterns indicating the abundance of CaZymes that have a Signal peptide relative to the abundance of each MAG in hylon

Supplementary figure 18

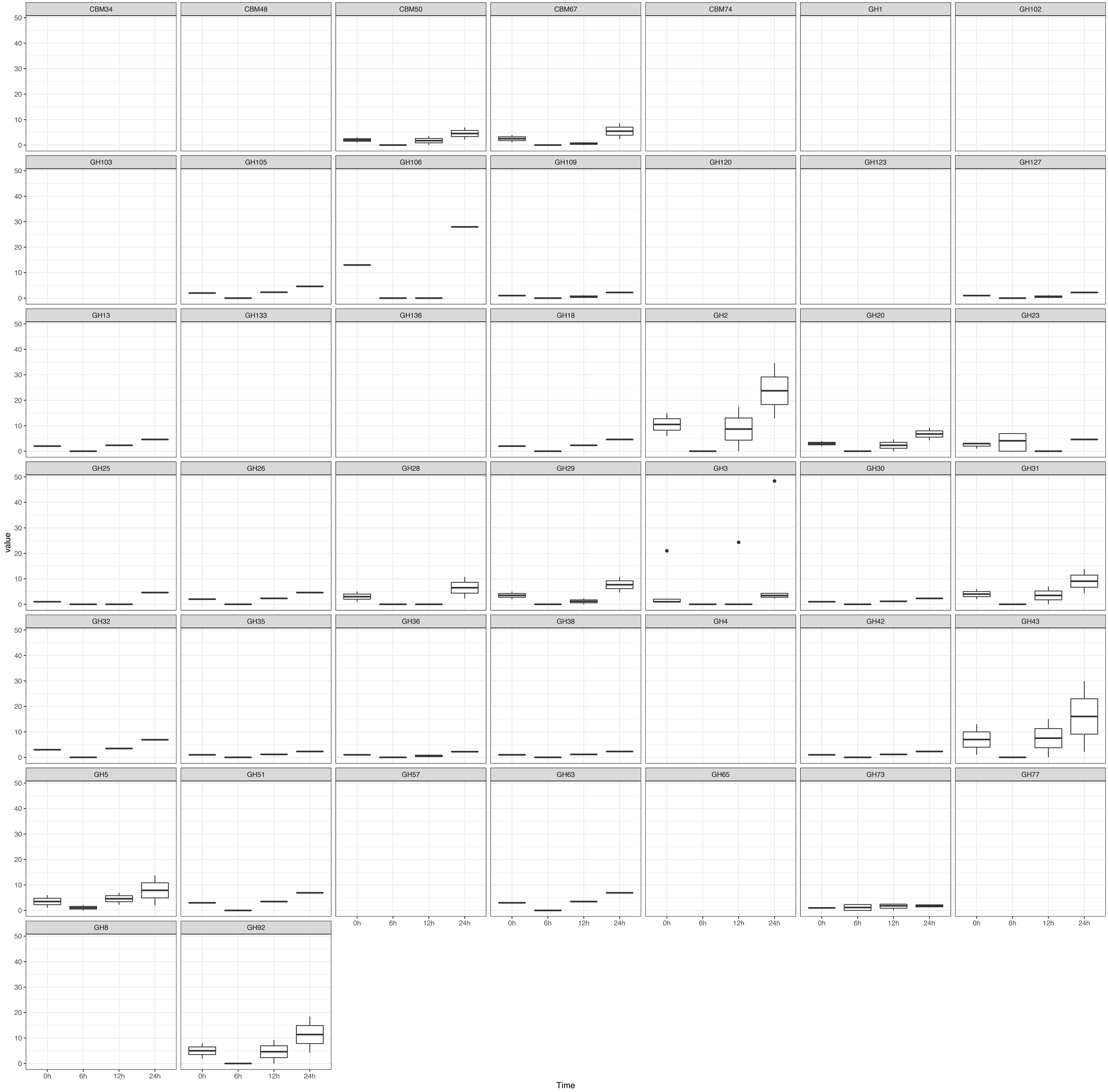

Supplementary figure 18: Patterns indicating the abundance of CaZymes that have a Signal peptide relative to the abundance of each MAG in inulin

Supplementary figure 19

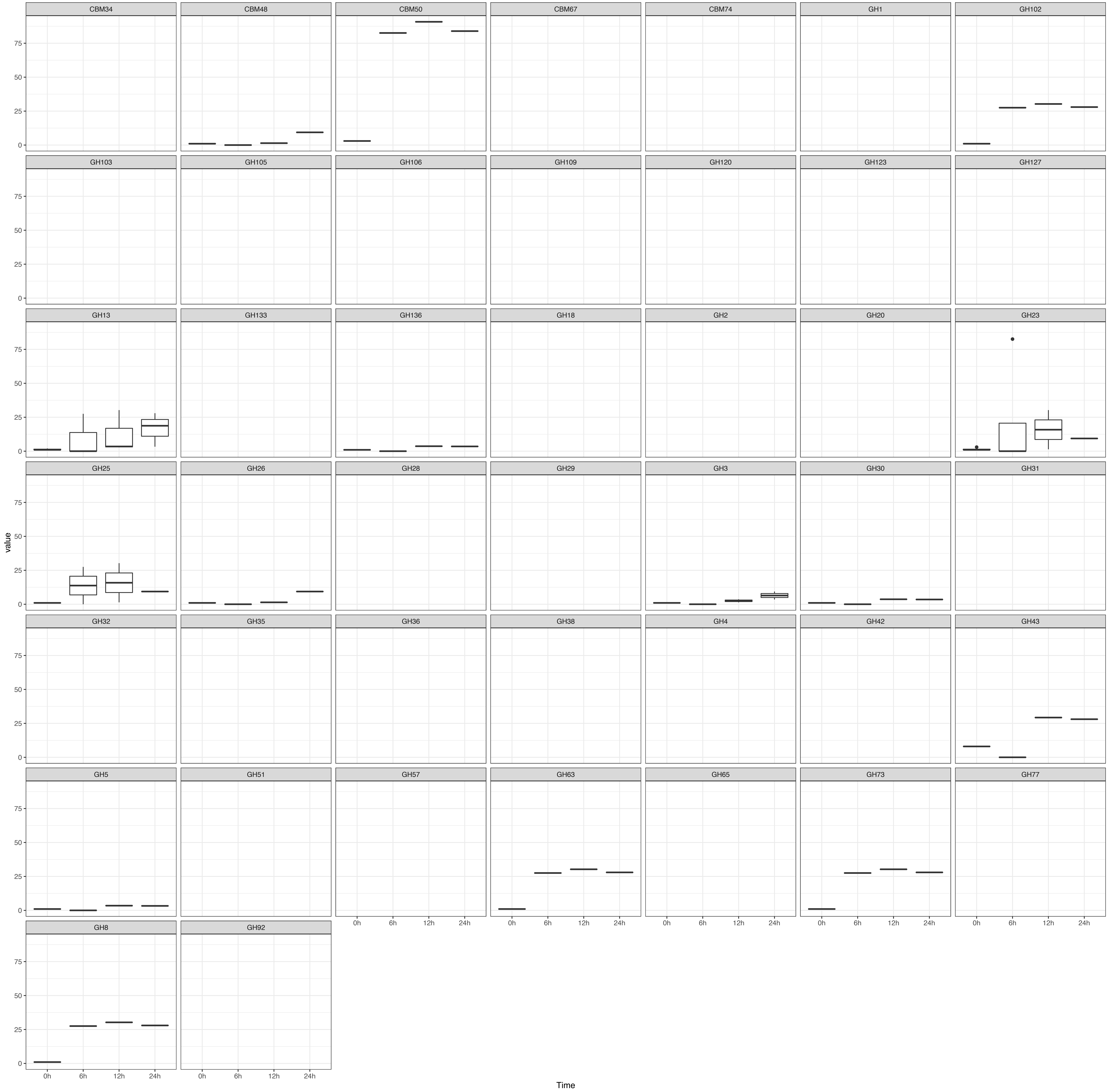

Supplementary figure 19: Patterns indicating the abundance of CaZymes that have a Signal peptide relative to the abundance of each MAG in normal maize

Supplementary figure 20

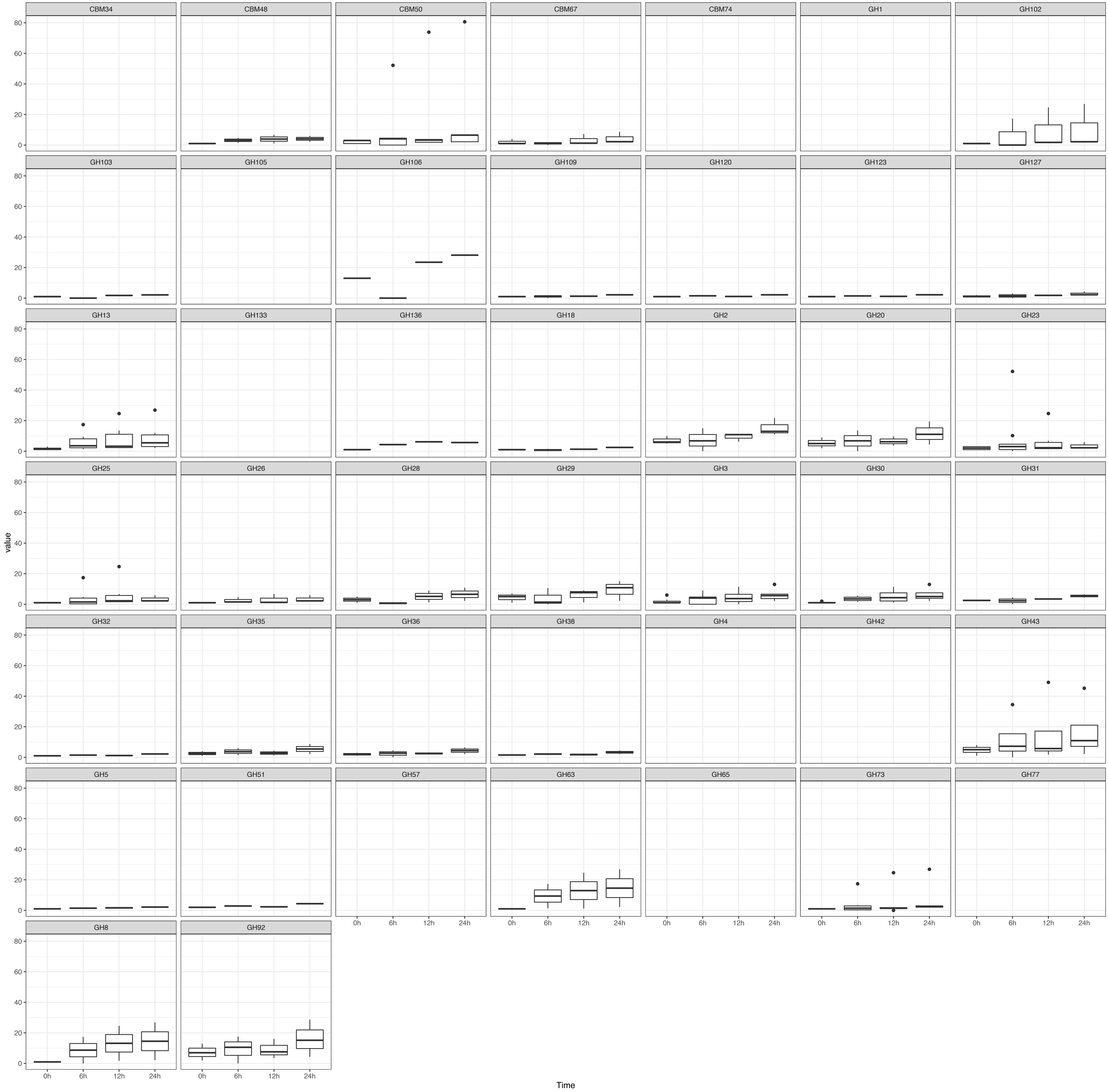

Supplementary figure 20: Patterns indicating the abundance of CaZymes that have a Signal peptide relative to the abundance of each MAG in retrograded maize

Supplementary figure 21

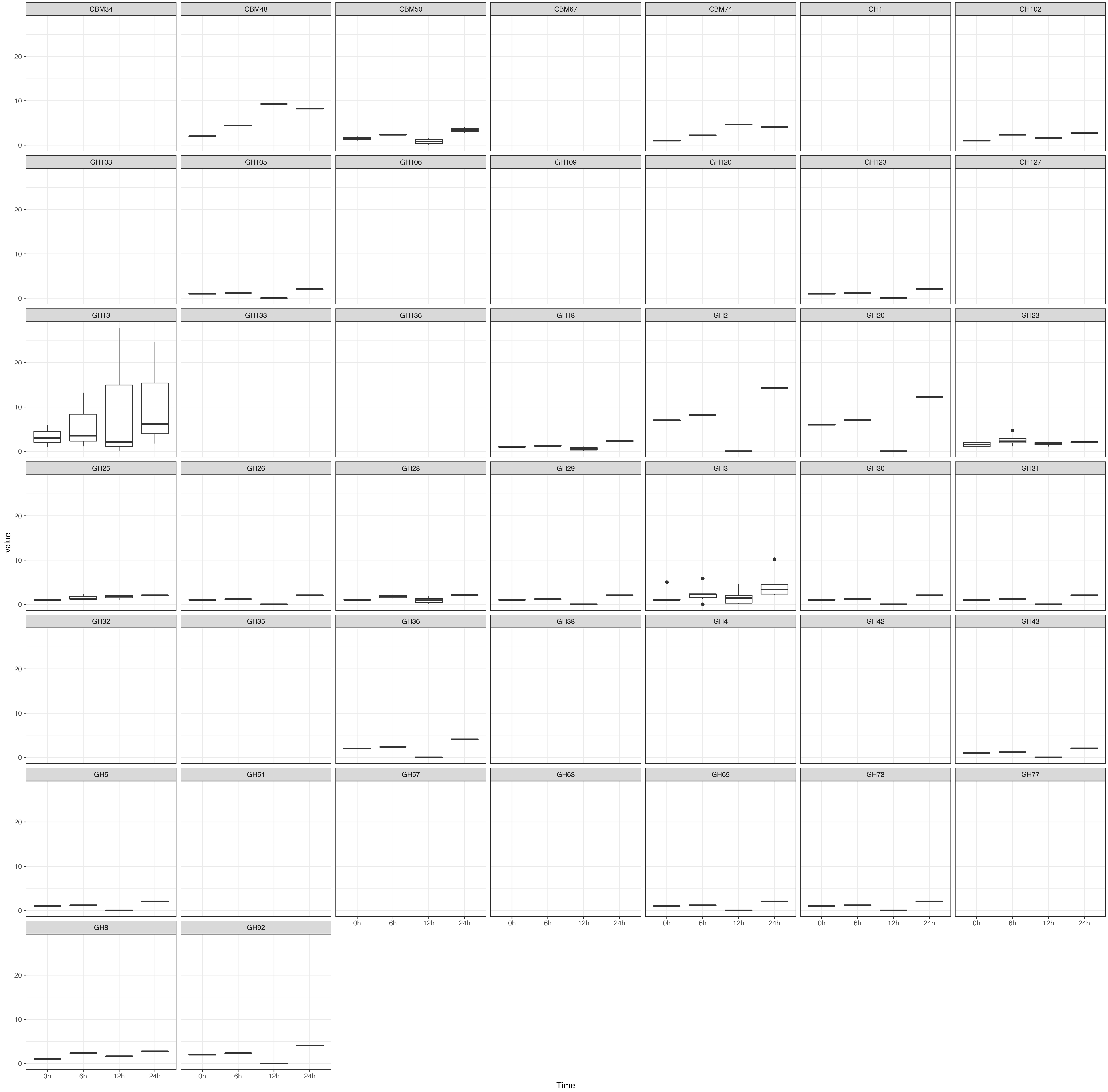

Supplementary figure 21: Patterns indicating the abundance of CaZymes that have a Signal peptide relative to the abundance of each MAG in potato

Supplementary Note 1- Latin names for MAGs not previous identified in NCBI

Description of *Candidatus* Acetatifactor hominis sp. nov.
Candidatus Acetatifactor hominis (ho'mi.nis. L. gen. masc. n. *hominis*, of a human being).

A bacterial species identified by metagenomic analyses. This species includes all bacteria with genomes that show ≥95% average nucleotide identity to the type genome for the species to which we have assigned the genome identifier rmaize_MAXBIN__038 and which is available via NCBI BioSample SAMN18871269. This is a new name for the alphanumeric GTDB species sp900066565. The GC content of the type genome is 47.74 % and the genome length is 3.05 Mbp.

Description of *Candidatus* Aphodonaster gen. nov.
Candidatus Aphodonaster (Aph.od.o.nas'ter. Gr. fem. n. *aphodos* dung; Gr. masc. n. *naster* an inhabitant; N.L. masc. n. *Aphodonaster* a microbe associated with faeces).

A bacterial genus identified by metagenomic analyses of human faeces. The genus includes all bacteria with genomes that show ≥60% average aminoacid identity to the genome of the type strain from the type species, *Candidatus* Aphodonaster merdae. This is a new name for the GTDB alphanumeric genus SFFH01. This genus has been assigned by GTDB-Tk v1.5.0 working on GTDB R06-RS202 reference data[1, 2] to the order *Christensenellales* and to the family *CAG-74*

Description of *Candidatus* Aphodonaster intestinalis sp. nov.
Candidatus Aphodonaster intestinalis (in.tes.ti.na'lis. N.L. masc. adj. *intestinalis,* pertaining to the intestines).

A bacterial species identified by metagenomic analyses. This species includes all bacteria with genomes that show ≥95% average nucleotide identity to the type genome for the species to which we have assigned the genome identifier T0_METABAT__97 and which is available via NCBI BioSample SAMN18871333. This is a new name for the alphanumeric GTDB species sp900548125. The GC content of the type genome is 55.44 % and the genome length is 2.54 Mbp.

Description of *Candidatus* Aphodonaster merdae sp. nov.
Candidatus Aphodonaster merdae (mer'dae. L. gen. fem. n. *merdae*, of faeces).

A bacterial species identified by metagenomic analyses. This species includes all bacteria with genomes that show ≥95% average nucleotide identity to the type genome for the species to which we have assigned the genome identifier pstarch_METABAT__69 and which is available via NCBI BioSample SAMN18871262. This is a new name for the alphanumeric GTDB species sp900542395. The GC content of the type genome is 59.61 % and the genome length is 2.66 Mbp.

Description of *Candidatus* Avimicrobium caecorum sp. nov.
Candidatus Avimicrobium caecorum (cae.co'rum. N. L. gen. pl. n. *caecorum*, of caeca).

A bacterial species identified by metagenomic analyses. This species includes all bacteria with genomes that show ≥95% average nucleotide identity to the type genome for the species to which we have assigned the genome identifier avicell_METABAT__34 and which is available via NCBI BioSample SAMN18871193. This is a new name for the alphanumeric GTDB species sp900547185. This genus was named by Glendinning et al. (2020). The GC content of the type genome is 56.83 % and the genome length is 2.20 Mbp.

Description of *Candidatus* Blautia hennigii sp. nov.
Candidatus Blautia hennigii (hen.ni'gi.i. N.L. gen. masc. n. *hennigii* derived from the Latinised family name for Willi Hennig, 1913-1976, the East German scientist who founded phylogenetic systematics or cladistics).

A bacterial species identified by metagenomic analyses. This species includes all bacteria with genomes that show ≥95% average nucleotide identity to the type genome for the species to which we have assigned the genome identifier hylon_METABAT__127 and which is available via NCBI BioSample SAMN18871203. This is a new name for the alphanumeric GTDB species sp900066505. GTDB has assigned this species to genus with an alphabetic suffix which cannot be incorporated into a well-formed binomial, so in naming this species, we have used the basonym for the genus. The GC content of the type genome is 43.26 % and the genome length is 2.93 Mbp.

Description of *Candidatus* Caccadaptatus gen. nov.
Candidatus Caccadaptatus (Cacc.ad.ap.ta'tus. Gr. fem. n. *kakké* dung; L. masc. part. adj. *adaptatus* adapted to; N.L. masc. n. *Caccadaptatus* a microbe associated with faeces).

A bacterial genus identified by metagenomic analyses of human faeces. The genus includes all bacteria with genomes that show ≥60% average aminoacid identity to the genome of the type strain from the type species, *Candidatus* Caccadaptatus darwinii. This is a new name for the GTDB alphanumeric genus NK3B98. This genus has been assigned by GTDB-Tk v1.5.0 working on GTDB R06-RS202 reference data[1, 2] to the order *Oscillospirales* and to the family *Oscillospiraceae*

Description of *Candidatus* Caccadaptatus darwinii sp. nov.
Candidatus Caccadaptatus darwinii (dar.wi'ni.i. N.L. gen. masc. n. darwinii derived from the Latinised family name for Charles Darwin, 1809-1882, the British scientist who proposed the theory of evolution by natural selection).

A bacterial species identified by metagenomic analyses. This species includes all bacteria with genomes that show ≥95% average nucleotide identity to the type genome for the species to which we have assigned the genome identifier rmaize_METABAT__56 and which is available via NCBI BioSample SAMN18871284. This is a new name for the alphanumeric GTDB species sp900545815. The GC content of the type genome is 56.11 % and the genome length is 2.31 Mbp.

Description of *Candidatus* Chesmatocola gen. nov.

Candidatus Chesmatocola (Ches.ma.to'co.la. Gr. neut. n. *chesma* dung; N.L. masc./fem. suffix *cola* an inhabitant of; N.L. fem. n. *Chesmatocola* a microbe associated with faeces).

A bacterial genus identified by metagenomic analyses of human faeces. The genus includes all bacteria with genomes that show ≥60% average aminoacid identity to the genome of the type strain from the type species, *Candidatus* Chesmatocola anthropi. This is a new name for the GTDB alphanumeric genus CAG-354. This genus has been assigned by GTDB-Tk v1.5.0 working on GTDB R06-RS202 reference data[1, 2] to the order *TANB77* and to the family *CAG-508*

Description of *Candidatus* Chesmatocola anthropi sp. nov.
Candidatus Chesmatocola anthropi (an.thro'pi. Gr. masc. n. *anthropos,* a human being; N.L. gen. masc. n. *anthropi,* of a human being).

A bacterial species identified by metagenomic analyses. This species includes all bacteria with genomes that show ≥95% average nucleotide identity to the type genome for the species to which we have assigned the genome identifier hylon_METABAT__79 and which is available via NCBI BioSample SAMN18871215. This is a new name for the alphanumeric GTDB species sp001915925. The GC content of the type genome is 28.31 % and the genome length is 1.38 Mbp.

Description of *Candidatus* Cholicomonas gen. nov.
Candidatus Cholicomonas (Cho.li.co.mo'nas. Gr. fem. n. *cholix, cholikos* guts; L. fem. n. *monas* a monad; N.L. fem. n. *Cholicomonas* a microbe associated with the intestines).

A bacterial genus identified by metagenomic analyses of human faeces. The genus includes all bacteria with genomes that show ≥60% average aminoacid identity to the genome of the type strain from the type species, *Candidatus* Cholicomonas copri. This is a new name for the GTDB alphanumeric genus CAG-628. This genus has been assigned by GTDB-Tk v1.5.0 working on GTDB R06-RS202 reference data[1, 2] to the order *RF39* and to the family *UBA660*

Description of *Candidatus* Cholicomonas copri sp. nov.
Candidatus Cholicomonas copri (cop'ri. Gr. masc. n. *kópros*, faeces; N.L. gen. n. copri; of faeces).

A bacterial species identified by metagenomic analyses. This species includes all bacteria with genomes that show ≥95% average nucleotide identity to the type genome for the species to which we have assigned the genome identifier T0_METABAT__30 and which is available via NCBI BioSample SAMN18871328. This is a new name for the alphanumeric GTDB species sp000438415. The GC content of the type genome is 27.35 % and the genome length is 0.62 Mbp.

Description of *Candidatus* Choliconaster gen. nov.
Candidatus Choliconaster (Cho.li.co.nas'ter. Gr. fem. n. *cholix, cholikos*guts; Gr. masc. n. *naster* an inhabitant; N.L. masc. n. *Choliconaster* a microbe associated with the intestines).

A bacterial genus identified by metagenomic analyses of human faeces. The genus includes all bacteria with genomes that show ≥60% average aminoacid identity to the genome of the type strain from the type species, *Candidatus* Choliconaster caccae. This is a new name for the GTDB alphanumeric genus ER4. This genus has been assigned by GTDB-Tk v1.5.0 working on GTDB R06-RS202 reference data[1, 2] to the order *Oscillospirales* and to the family *Oscillospiraceae*

Description of *Candidatus* Choliconaster caccae sp. nov.
Candidatus Choliconaster caccae (cac'cae. Gr. fem. n. *kakkê*, faeces; N.L. gen. n. *caccae*, of faeces).

A bacterial species identified by metagenomic analyses. This species includes all bacteria with genomes that show ≥95% average nucleotide identity to the type genome for the species to which we have assigned the genome identifier T0_MAXBIN__028 and which is available via NCBI BioSample SAMN18871294. This is a new name for the alphanumeric GTDB species sp000765235. The GC content of the type genome is 57.68 % and the genome length is 2.86 Mbp.

Description of *Candidatus* Choliconaster merdae sp. nov.
Candidatus Choliconaster merdae (mer'dae. L. gen. fem. n. *merdae*, of faeces).

A bacterial species identified by metagenomic analyses. This species includes all bacteria with genomes that show ≥95% average nucleotide identity to the type genome for the species to which we have assigned the genome identifier T0_METABAT__240 and which is available via NCBI BioSample SAMN18871322. This is a new name for the alphanumeric GTDB species sp900317525. The GC content of the type genome is 60.79 % and the genome length is 1.92 Mbp.

Description of *Candidatus* Colibacterium gen. nov.
Candidatus Colibacterium (Co.li.bac.te'ri.um. L. neut. n. *colon* large intestine; N.L. neut. n. *bacterium* a bacterium; N.L. neut. n. *Colibacterium* a microbe associated with the intestines).

A bacterial genus identified by metagenomic analyses of human faeces. The genus includes all bacteria with genomes that show ≥60% average aminoacid identity to the genome of the type strain from the type species, *Candidatus* Colibacterium hominis. This is a new name for the GTDB alphanumeric genus SFEL01. This genus has been assigned by GTDB-Tk v1.5.0 working on GTDB R06-RS202 reference data[1, 2] to the order *Christensenellales* and to the family *CAG-138*

Description of *Candidatus* Colibacterium hominis sp. nov.
Candidatus Colibacterium hominis (ho'mi.nis. L. gen. masc. n. *hominis*, of a human being).

A bacterial species identified by metagenomic analyses. This species includes all bacteria with genomes that show ≥95% average nucleotide identity to the type genome for the species to which we have assigned the genome identifier T0_MAXBIN__134 and which is available via NCBI BioSample SAMN18871298. This is a new name for the alphanumeric GTDB species sp004557245. The GC content of the type genome is 54.28 % and the genome length is 1.56 Mbp.

Description of *Candidatus* Colihabitans gen. nov.
Candidatus Colihabitans (Co.li.ha'bi.tans. L. neut. n. *colon* large intestine; L. masc./fem. adj. part. *habitans* an inhabitant; N.L. fem. n. *Colihabitans* a microbe associated with the intestines).

A bacterial genus identified by metagenomic analyses of human faeces. The genus includes all bacteria with genomes that show ≥60% average aminoacid identity to the genome of the type strain from the type species, *Candidatus* Colihabitans norwichensis. This is a new name for the GTDB alphanumeric genus CAG-170. This genus has been assigned by GTDB-Tk v1.5.0 working on GTDB R06-RS202 reference data[1, 2] to the order *Oscillospirales* and to the family *Oscillospiraceae*

Description of *Candidatus* Colihabitans hominis sp. nov.
Candidatus Colihabitans hominis (ho'mi.nis. L. gen. masc. n. *hominis*, of a human being).

A bacterial species identified by metagenomic analyses. This species includes all bacteria with genomes that show ≥95% average nucleotide identity to the type genome for the species to which we have assigned the genome identifier T0_METABAT__180 and which is available via NCBI BioSample SAMN18871312. This is a new name for the alphanumeric GTDB species sp900549635. The GC content of the type genome is 56.47 % and the genome length is 2.50 Mbp.

Description of *Candidatus* Colihabitans norwichensis sp. nov.
Candidatus Colihabitans norwichensis (nor.wich.en'sis. N.L. fem. adj. *norwichensis* pertaining to English city of Norwich).

A bacterial species identified by metagenomic analyses. This species includes all bacteria with genomes that show ≥95% average nucleotide identity to the type genome for the species to which we have assigned the genome identifier hylon_METABAT__172 and which is available via NCBI BioSample SAMN18871205. This is a new name for the alphanumeric GTDB species sp000432135. The GC content of the type genome is 57.56 % and the genome length is 3.16 Mbp.

Description of *Candidatus* Dysosmobacter stercoris sp. nov.
Candidatus Dysosmobacter stercoris (ster'co.ris. L. gen. neut. n. *stercoris*, of faeces).

A bacterial species identified by metagenomic analyses. This species includes all bacteria with genomes that show ≥95% average nucleotide identity to the type genome for the species to which we have assigned the genome identifier hylon_METABAT__95 and which is available via NCBI BioSample SAMN18871217. This is a new name for the alphanumeric GTDB species sp900542115. The GC content of the type genome is 58.43 % and the genome length is 1.44 Mbp.

Description of *Candidatus* Eisenbergiella faecalis sp. nov.
Candidatus Eisenbergiella faecalis (fae.ca'lis. N.L. fem. adj. *faecalis*, of faeces).

A bacterial species identified by metagenomic analyses. This species includes all bacteria with genomes that show ≥95% average nucleotide identity to the type genome for the species to which we have assigned the genome identifier

pstarch_METABAT__22 and which is available via NCBI BioSample SAMN18871259. This is a new name for the alphanumeric GTDB species sp900066775. The GC content of the type genome is 48.80 % and the genome length is 2.82 Mbp.

Description of *Candidatus* Enteromorpha gen. nov.
Candidatus Enteromorpha (En.te.ro.mor'pha. Gr. neut. n. *enteron* the gut; Gr. fem. n. *morphe* a form, shape; N.L. fem. n. *Enteromorpha* a microbe associated with the intestines).

A bacterial genus identified by metagenomic analyses of human faeces. The genus includes all bacteria with genomes that show ≥60% average aminoacid identity to the genome of the type strain from the type species, *Candidatus* Enteromorpha quadrami. This is a new name for the GTDB alphanumeric genus CAG-110. This genus has been assigned by GTDB-Tk v1.5.0 working on GTDB R06-RS202 reference data[1, 2] to the order *Oscillospirales* and to the family *Oscillospiraceae*

Description of *Candidatus* Enteromorpha barnesiae sp. nov.
Candidatus Enteromorpha barnesiae (bar.ne'si.ae. N.L. gen. fem. n. *barnesiae*, of Barnes, named after Ella M. Barnes, a British microbiologist).

A bacterial species identified by metagenomic analyses. This species includes all bacteria with genomes that show ≥95% average nucleotide identity to the type genome for the species to which we have assigned the genome identifier avicell_METABAT__70, hylon_METABAT__9, inulin_METABAT__82 and rmaize_METABAT__177 and which is available via NCBI BioSample SAMN18871197. This is a new name for the alphanumeric GTDB species sp003525905. The GC content of the type genome is 61.70 %, 61.32 %, 61.45 % and 62.01 % and the genome length is 1.70 Mbp, 2.26 Mbp, 2.12 Mbp and 1.81 Mbp.

Description of *Candidatus* Enteromorpha quadrami sp. nov.
Candidatus Enteromorpha quadrami (quad.ra'mi. N.L. gen. n. *quadrami* of the Quadram Institute).

A bacterial species identified by metagenomic analyses. This species includes all bacteria with genomes that show ≥95% average nucleotide identity to the type genome for the species to which we have assigned the genome identifiers avicel_MAXBIN__045, inulin_METABAT__94 and pstarch_METABAT__151 and which is available via NCBI BioSample SAMN18871185. This is a new name for the alphanumeric GTDB species sp000434635. The GC content of the type genome is 57.48 %, 57.06 % and 57.30 5 and the genome length are 2.09 Mbp, 2.31 Mbp and 2.27 Mbp.

Description of *Candidatus* Enteronaster gen. nov.
Candidatus Enteronaster (En.ter.o.nas'ter. Gr. neut. n. *enteron* the gut; Gr. masc. n. *naster* an inhabitant; N.L. masc. n. *Enteronaster* a microbe associated with the intestines).

A bacterial genus identified by metagenomic analyses of human faeces. The genus includes all bacteria with genomes that show ≥60% average aminoacid identity to the genome of the type strain from the type species, *Candidatus* Enteronaster faecalis.

This is a new name for the GTDB alphanumeric genus CAG-103. This genus has been assigned by GTDB-Tk v1.5.0 working on GTDB R06-RS202 reference data [1, 2]to the order *Oscillospirales* and to the family *Oscillospiraceae*

Description of *Candidatus* Enteronaster faecalis sp. nov.
Candidatus Enteronaster faecalis (fae.ca'lis. N.L. masc. adj. *faecalis*, of faeces).

A bacterial species identified by metagenomic analyses. This species includes all bacteria with genomes that show ≥95% average nucleotide identity to the type genome for the species to which we have assigned the genome identifier inulin_METABAT__98 and which is available via NCBI BioSample SAMN18871242. This is a new name for the alphanumeric GTDB species sp000432375. The GC content of the type genome is 61.98 % and the genome length is 1.97 Mbp.

Description of *Candidatus* Enteroplasma gen. nov.
Candidatus Enteroplasma (En.te.ro.plas'ma. Gr. neut. n. *enteron* the gut; L. neut. n. *plasma* a form; N.L. neut. n. *Enteroplasma* a microbe associated with the intestines).

A bacterial genus identified by metagenomic analyses of human faeces. The genus includes all bacteria with genomes that show ≥60% average aminoacid identity to the genome of the type strain from the type species, *Candidatus* Enteroplasma stercoris. This is a new name for the GTDB alphanumeric genus CAG-115. This genus has been assigned by GTDB-Tk v1.5.0 working on GTDB R06-RS202 reference data[1, 2] to the order *Oscillospirales* and to the family *Ruminococcaceae*

Description of *Candidatus* Enteroplasma stercoris sp. nov.
Candidatus Enteroplasma stercoris (ster'co.ris. L. gen. neut. n. *stercoris*, of faeces).

A bacterial species identified by metagenomic analyses. This species includes all bacteria with genomes that show ≥95% average nucleotide identity to the type genome for the species to which we have assigned the genome identifier inulin_MAXBIN__035 and which is available via NCBI BioSample SAMN18871220. This is a new name for the alphanumeric GTDB species sp003531585. The GC content of the type genome is 52.91 % and the genome length is 2.79 Mbp.

Description of *Candidatus* Enterovivens gen. nov.
Candidatus Enterovivens (En.te.ro.vi'vens. Gr. neut. n. *enteron* the gut; N.L. masc./fem. adj. part. *vivens* living; N.L. fem. n. *Enterovivens* a microbe living in the intestines).

A bacterial genus identified by metagenomic analyses of human faeces. The genus includes all bacteria with genomes that show ≥60% average aminoacid identity to the genome of the type strain from the type species, *Candidatus* Enterovivens caccae. This is a new name for the GTDB alphanumeric genus CAG-127. This genus has been assigned by GTDB-Tk v1.5.0 working on GTDB R06-RS202 reference data[1, 2] to the order *Lachnospirales* and to the family *Lachnospiraceae*

Description of *Candidatus* Enterovivens caccae sp. nov.
Candidatus Enterovivens caccae (cac'cae. Gr. fem. n. *kakkê*, faeces; N.L. gen. n. *caccae*, of faeces).

A bacterial species identified by metagenomic analyses. This species includes all bacteria with genomes that show ≥95% average nucleotide identity to the type genome for the species to which we have assigned the genome identifier T0_METABAT__183 and which is available via NCBI BioSample SAMN18871313. This is a new name for the alphanumeric GTDB species sp900319515. The GC content of the type genome is 44.48 % and the genome length is 2.61 Mbp.

Description of *Candidatus* Eubacterium caccanthorpi sp. nov.
Candidatus Eubacterium caccanthorpi (cacc.an.thro'pi. Gr. fem. n. *kakkê*, faeces; Gr. masc. n. *anthropos*, a human being; N.L. gen. masc. n. *caccanthropi*, of human faeces).

A bacterial species identified by metagenomic analyses. This species includes all bacteria with genomes that show ≥95% average nucleotide identity to the type genome for the species to which we have assigned the genome identifier T0_MAXBIN__022 and which is available via NCBI BioSample SAMN18871293. This is a new name for the alphanumeric GTDB species sp000434995. GTDB has assigned this species to genus with an alphabetic suffix which cannot be incorporated into a well-formed binomial, so in naming this species, we have used the basonym for the genus. The GC content of the type genome is 36.52 % and the genome length is 1.94 Mbp.

Description of *Candidatus* Eubacterium colihabitans sp. nov.
Candidatus Eubacterium colihabitans (co.li.ha'bi.tans. L. neut. n. *colum*, colon; L. pres. part. *habitans*, inhabiting; N.L. part. adj. *colihabitans*, inhabiting the colon).

A bacterial species identified by metagenomic analyses. This species includes all bacteria with genomes that show ≥95% average nucleotide identity to the type genome for the species to which we have assigned the genome identifier T0_METABAT__220 and which is available via NCBI BioSample SAMN18871319. This is a new name for the alphanumeric GTDB species sp003491505. GTDB has assigned this species to genus with an alphabetic suffix which cannot be incorporated into a well-formed binomial, so in naming this species, we have used the basonym for the genus. The GC content of the type genome is 41.07 % and the genome length is 2.49 Mbp.

Description of *Candidatus* Gallacutalibacter hominis sp. nov.
Candidatus Gallacutalibacter hominis (ho'mi.nis. L. gen. masc. n. *hominis*, of a human being).

A bacterial species identified by metagenomic analyses. This species includes all bacteria with genomes that show ≥95% average nucleotide identity to the type genome for the species to which we have assigned the genome identifiers avicel__METABAT__20 and inulin_METABAT__180 and which is available via NCBI BioSample SAMN18871192. This is a new name for the alphanumeric GTDB species sp003477405. This genus was named by Gilroy et al. (2021). The GC content of the type genomes is 56.15 % and 56. 25 % and the genome lengths are 2.33 Mbp and 1.92 Mbp.

Description of *Candidatus* Gemmiger merdicola sp. nov.

Candidatus Gemmiger merdicola (mer.di'co.la. L. gen. fem. n. *merda*, faeces; L. masc./fem. suff. *-cola*, inhabitant of; N.L. fem. n. *merdicola* inhabitant of faeces).

A bacterial species identified by metagenomic analyses. This species includes all bacteria with genomes that show ≥95% average nucleotide identity to the type genome for the species to which we have assigned the genome identifier inulin_METABAT__93 and which is available via NCBI BioSample SAMN18871240. This is a new name for the alphanumeric GTDB species sp900539695. The GC content of the type genome is 58.43 % and the genome length is 2.39 Mbp.

Description of *Candidatus* Huxleyella gen. nov.
Candidatus Huxleyella (Hux.ley.el'la. L. fem. dim. suff. *-ella* diminutive ending; N.L. fem. n. *Huxleyella* named in honour of the British scientist Thomas Henry Huxley (1825-1895), known for his advocacy of Charles Darwin's theory of evolution).

A bacterial genus identified by metagenomic analyses of human faeces. The genus includes all bacteria with genomes that show ≥60% average aminoacid identity to the genome of the type strain from the type species, *Candidatus* Huxleyella fimi. This is a new name for the GTDB alphanumeric genus UMGS1071. This genus has been assigned by GTDB-Tk v1.5.0 working on GTDB R06-RS202 reference data [1, 2]to the order *Oscillospirales* and to the family *Acutalibacteraceae*

Description of *Candidatus* Huxleyella fimi sp. nov.
Candidatus Huxleyella fimi (fi'mi. L. neut. gen. n. *fimi*, of faeces).

A bacterial species identified by metagenomic analyses. This species includes all bacteria with genomes that show ≥95% average nucleotide identity to the type genome for the species to which we have assigned the genome identifier T0_METABAT__173 and which is available via NCBI BioSample SAMN18871311. This is a new name for the alphanumeric GTDB species sp900542375. The GC content of the type genome is 38.84 % and the genome length is 1.60 Mbp.

Description of *Candidatus* Minthomorpha gen. nov.
Candidatus Minthomorpha (Min.tho.mor'pha. Gr. masc. n. *minthos* dung; Gr. fem. n. *morphe* a form, shape; N.L. fem. n. *Minthomorpha* a microbe associated with faeces).

A bacterial genus identified by metagenomic analyses of human faeces. The genus includes all bacteria with genomes that show ≥60% average aminoacid identity to the genome of the type strain from the type species, *Candidatus* Minthomorpha faecalis. This is a new name for the GTDB alphanumeric genus CAG-81. This genus has been assigned by GTDB-Tk v1.5.0 working on GTDB R06-RS202 reference data[1, 2] to the order *Lachnospirales* and to the family *Lachnospiraceae*

Description of *Candidatus* Minthomorpha faecalis sp. nov.
Candidatus Minthomorpha faecalis (fae.ca'lis. N.L. fem. adj. *faecalis*, of faeces).

A bacterial species identified by metagenomic analyses. This species includes all bacteria with genomes that show ≥95% average nucleotide identity to the type genome for the species to which we have assigned the genome identifier rmaize_METABAT__174 and which is available via NCBI BioSample

SAMN18871281. This is a new name for the alphanumeric GTDB species sp900066535. The GC content of the type genome is 49.05 % and the genome length is 2.98 Mbp.

Description of *Candidatus* Minthonaster gen. nov.
Candidatus Minthonaster (Min.tho.nas'ter. Gr. masc. n. *minthos* dung; Gr. masc. n. *naster* an inhabitant; N.L. masc. n. *Minthonaster* a microbe associated with faeces).

A bacterial genus identified by metagenomic analyses of human faeces. The genus includes all bacteria with genomes that show ≥60% average aminoacid identity to the genome of the type strain from the type species, *Candidatus* Minthonaster faecium. This is a new name for the GTDB alphanumeric genus CAG-83. This genus has been assigned by GTDB-Tk v1.5.0 working on GTDB R06-RS202 reference data[1, 2] to the order *Oscillospirales* and to the family *Oscillospiraceae*

Description of *Candidatus* Minthonaster anthropi sp. nov.
Candidatus Minthonaster anthropi (an.thro'pi. Gr. masc. n. *anthropos,* a human being; N.L. gen. masc. n. *anthropi*, of a human being).

A bacterial species identified by metagenomic analyses. This species includes all bacteria with genomes that show ≥95% average nucleotide identity to the type genome for the species to which we have assigned the genome identifier T0_METABAT__167 and which is available via NCBI BioSample SAMN18871310. This is a new name for the alphanumeric GTDB species sp900552475. The GC content of the type genome is 61.38 % and the genome length is 2.18 Mbp.

Description of *Candidatus* Minthonaster faecium sp. nov.
Candidatus Minthonaster faecium (fae'ci.um. L. fem. gen. pl. n. *faecium,* of faeces).

A bacterial species identified by metagenomic analyses. This species includes all bacteria with genomes that show ≥95% average nucleotide identity to the type genome for the species to which we have assigned the genome identifier hylon_METABAT__44 and which is available via NCBI BioSample SAMN18871213. This is a new name for the alphanumeric GTDB species sp003539495. The GC content of the type genome is 57.03 % and the genome length is 2.06 Mbp.

Description of *Candidatus* Minthonaster hominis sp. nov.
Candidatus Minthonaster hominis (ho'mi.nis. L. gen. masc. n. *hominis*, of a human being).

A bacterial species identified by metagenomic analyses. This species includes all bacteria with genomes that show ≥95% average nucleotide identity to the type genome for the species to which we have assigned the genome identifier inulin_METABAT__175 and which is available via NCBI BioSample SAMN18871228. This is a new name for the alphanumeric GTDB species sp900545585. The GC content of the type genome is 60.55 % and the genome length is 2.24 Mbp.

Description of *Candidatus* Minthonaster merdae sp. nov.
Candidatus Minthonaster merdae (mer'dae. L. gen. fem. n. *merdae*, of faeces).

A bacterial species identified by metagenomic analyses. This species includes all bacteria with genomes that show ≥95% average nucleotide identity to the type genome for the species to which we have assigned the genome identifier T0_METABAT__66 and which is available via NCBI BioSample SAMN18871332. This is a new name for the alphanumeric GTDB species sp000431575. The GC content of the type genome is 59.89 % and the genome length is 2.00 Mbp.

Description of *Candidatus* Minthoplasma gen. nov.
Candidatus Minthoplasma (Min.tho.plas'ma. Gr. masc. n. *minthos* dung; L. neut. n. *plasma* a form; *Minthoplasma* a microbe associated with faeces).

A bacterial genus identified by metagenomic analyses of human faeces. The genus includes all bacteria with genomes that show ≥60% average aminoacid identity to the genome of the type strain from the type species, *Candidatus* Minthoplasma entericum. This is a new name for the GTDB alphanumeric genus GCA-900066135. This genus has been assigned by GTDB-Tk v1.5.0 working on GTDB R06-RS202 reference data[1, 2] to the order *Lachnospirales* and to the family *Lachnospiraceae*

Description of *Candidatus* Minthoplasma copri sp. nov.
Candidatus Minthoplasma copri (cop'ri. Gr. masc. n. kópros, faeces; N.L. gen. n. copri; of faeces).

A bacterial species identified by metagenomic analyses. This species includes all bacteria with genomes that show ≥95% average nucleotide identity to the type genome for the species to which we have assigned the genome identifier T0_METABAT__206 and which is available via NCBI BioSample SAMN18871318. This is a new name for the alphanumeric GTDB species sp900543575. The GC content of the type genome is 49.81 % and the genome length is 3.26 Mbp.

Description of *Candidatus* Minthoplasma enterica sp. nov.
Candidatus Minthoplasma entericum (en.te'ri.cum. Gr. neut. n. *enteron*, gut; L. neut. adj. suff. *-icum*, pertaining to; N.L. neut. adj.*entericum*, pertaining to the gut).

A bacterial species identified by metagenomic analyses. This species includes all bacteria with genomes that show ≥95% average nucleotide identity to the type genome for the species to which we have assigned the genome identifier T0_METABAT__122 and which is available via NCBI BioSample SAMN18871303. This is a new name for the alphanumeric GTDB species sp900066135. The GC content of the type genome is 47.02 % and the genome length is 1.90 Mbp.

Description of *Candidatus* Minthovivens gen. nov.
Candidatus Minthovivens (Min.tho.viv'ens. Gr. masc. n. *minthos* dung; N.L. masc./fem. part. adj. *vivens* living; N.L. fem. n. *Minthovivens* a microbe living in faeces).

A bacterial genus identified by metagenomic analyses of human faeces. The genus includes all bacteria with genomes that show ≥60% average aminoacid identity to the genome of the type strain from the type species, *Candidatus* Minthovivens enterohominis. This is a new name for the GTDB alphanumeric genus KLE1615. This genus has been assigned by GTDB-Tk v1.5.0 working on GTDB R06-RS202 reference data[1, 2] to the order *Lachnospirales* and to the family *Lachnospiraceae*

Description of *Candidatus* Minthovivens enterohominis sp. nov.
Candidatus Minthovivens enterohominis (en.te.ro.ho'mi.nis. Gr. neut. n. *enteron*, gut; L. gen. masc. n. *hominis,* of a human being; N.L. gen. masc. n. *enterohominis*, of the human gut).

A bacterial species identified by metagenomic analyses. This species includes all bacteria with genomes that show ≥95% average nucleotide identity to the type genome for the species to which we have assigned the genome identifier inulin_METABAT__130 and which is available via NCBI BioSample SAMN18871226. This is a new name for the alphanumeric GTDB species sp900066985. The GC content of the type genome is 40.97 % and the genome length is 3.77 Mbp.

Description of *Candidatus* Negativibacillus quadrami sp. nov.
Candidatus Negativibacillus quadrami (quad.ra'mi. N.L. gen. n. *quadrami* of the Quadram Institute).

A bacterial species identified by metagenomic analyses. This species includes all bacteria with genomes that show ≥95% average nucleotide identity to the type genome for the species to which we have assigned the genome identifier T0_METABAT__114 and which is available via NCBI BioSample SAMN18871301. This is a new name for the alphanumeric GTDB species sp000435195. The GC content of the type genome is 51.95 % and the genome length is 2.20 Mbp.

Description of *Candidatus* Neoacutalibacter gen. nov.
Candidatus Neoacutalibacter (Ne.o.a.cu.ta.li.ibac'ter. Gr. masc. adj. *neos* new; N.L. masc. n. *Acutalibacter* an existing genus name; N.L. masc. n. *Neoacutalibacter* a bacterial genus related to but distinct from the existing named genus).

A bacterial genus identified by metagenomic analyses of human faeces. The genus includes all bacteria with genomes that show ≥60% average aminoacid identity to the genome of the type strain from the type species, *Candidatus* Neoacutalibacter hominis. This is a new name for the GTDB alphanumeric genus CAG-177. This genus has been assigned by GTDB-Tk v1.5.0 working on GTDB R06-RS202 reference data[1, 2] to the order *Oscillospirales* and to the family *Acutalibacteraceae*

Description of *Candidatus* Neoacutalibacter hominis sp. nov.
Candidatus Neoacutalibacter hominis (ho'mi.nis. L. gen. masc. n. *hominis*, of a human being).

A bacterial species identified by metagenomic analyses. This species includes all bacteria with genomes that show ≥95% average nucleotide identity to the type genome for the species to which we have assigned the genome identifier inulin_MAXBIN__022 and which is available via NCBI BioSample SAMN18871219. This is a new name for the alphanumeric GTDB species sp003514385. The GC content of the type genome is 51.47 % and the genome length is 2.22 Mbp.

Description of *Candidatus* Neoanaerovorax gen. nov.

Candidatus Neoanaerovorax (Ne.o.an.ae.ro.vo'rax. Gr. masc. adj. *neos* new; N.L. masc. n. *Anaerovorax* an existing genus name; N.L. masc. n. *Neoanaerovorax* a bacterial genus related to but distinct from the existing named genus).

A bacterial genus identified by metagenomic analyses of human faeces. The genus includes all bacteria with genomes that show ≥60% average aminoacid identity to the genome of the type strain from the type species, *Candidatus* Neoanaetovorax merdae. This is a new name for the GTDB alphanumeric genus CAG-238. This genus has been assigned by GTDB-Tk v1.5.0 working on GTDB R06-RS202 reference data[1, 2] to the order *Peptostreptococcales* and to the family *Anaerovoracaceae*

Description of *Candidatus* Neoanaerovorax merdae sp. nov.
Candidatus Neoanaerovorax merdae (mer'dae. L. gen. fem. n. *merdae*, of faeces).

A bacterial species identified by metagenomic analyses. This species includes all bacteria with genomes that show ≥95% average nucleotide identity to the type genome for the species to which we have assigned the genome identifiers rmaize_METABAT__46_sub and T0_METABAT__161 and which is available via NCBI BioSample SAMN18871283. This is a new name for the alphanumeric GTDB species sp900542245. The GC content of the type genome are 52.09 % and 51.52 % and the genome lengths are 1.57 Mbp and 2.01 Mbp.

Description of *Candidatus* Neoeggerthella gen. nov.
Candidatus Neoeggerthella (Ne.o.eg.ger.thel'la. Gr. masc. adj. *neos* new; N.L. fem. n. *Eggerthella* an existing genus name; N.L. fem. n. *Neoeggerthella* a bacterial genus related to but distinct from the existing named genus).

A bacterial genus identified by metagenomic analyses of human faeces. The genus includes all bacteria with genomes that show ≥60% average aminoacid identity to the genome of the type strain from the type species, *Candidatus* Neoeggerthella hominis. This is a new name for the GTDB alphanumeric genus CAG-1427. This genus has been assigned by GTDB-Tk v1.5.0 working on GTDB R06-RS202 reference data[1, 2] to the order *Coriobacteriales* and to the family *Eggerthellaceae*´

Description of *Candidatus* Neoeggerthella hominis sp. nov.
Candidatus Neoeggerthella hominis (ho'mi.nis. L. gen. masc. n. *hominis*, of a human being).

A bacterial species identified by metagenomic analyses. This species includes all bacteria with genomes that show ≥95% average nucleotide identity to the type genome for the species to which we have assigned the genome identifier nmaize_METABAT__52 and which is available via NCBI BioSample SAMN18871250. This is a new name for the alphanumeric GTDB species sp900554685. The GC content of the type genome is 45.89 % and the genome length is 1.92 Mbp.

Description of *Candidatus* Pararuminococcus gen. nov.
Candidatus Pararuminococcus (Pa.ra.ru.mi.no.coc'cus. Gr. pref. *para-* beside; N.L. masc. n. *Ruminococcus* an existing genus name; N.L. masc. n. *Pararuminococcus* a bacterial genus related to but distinct from the existing named genus).

A bacterial genus identified by metagenomic analyses of human faeces. The genus includes all bacteria with genomes that show ≥60% average aminoacid identity to the genome of the type strain from the type species, *Candidatus* Parauminococcus sangeri. This is a new name for the GTDB alphanumeric genus UBA1417. This genus has been assigned by GTDB-Tk v1.5.0 working on GTDB R06-RS202 reference data[1, 2] to the order *Oscillospirales* and to the family *Acutalibacteraceae*

Description of *Candidatus* Parasutterella caccanthropi sp. nov.
Candidatus Parasutterella caccanthropi (cacc.an.thro'pi. Gr. fem. n. *kakkê*, faeces; Gr. masc. n. *anthropos*, a human being; N.L. gen. masc. n. *caccanthropi*, of human faeces).

A bacterial species identified by metagenomic analyses. This species includes all bacteria with genomes that show ≥95% average nucleotide identity to the type genome for the species to which we have assigned the genome identifier pstarch_METABAT__57 and which is available via NCBI BioSample SAMN18871261. This is a new name for the alphanumeric GTDB species sp000980495. The GC content of the type genome is 49.32 % and the genome length is 2.19 Mbp.

Description of *Candidatus* Parauminococcus sangeri sp. nov.
Candidatus Parauminococcus sangeri (san'ge.ri. N.L. masc. n. *sangeri* derived from the Latinised family name for Frederick Sanger, 1918-2013, the British scientist; awarded the 1958 Nobel Prize in Chemistry for his work on the structure of protein and the 1980 Nobel Prize in Chemistry for inventing dideoxy sequencing).

A bacterial species identified by metagenomic analyses. This species includes all bacteria with genomes that show ≥95% average nucleotide identity to the type genome for the species to which we have assigned the genome identifier T0_METABAT__250 and which is available via NCBI BioSample SAMN18871325. This is a new name for the alphanumeric GTDB species sp003531055. The GC content of the type genome is 53.40 % and the genome length is 2.30 Mbp.

Description of *Candidatus* Pearsonella gen. nov.
Candidatus Pearsonella (Pear.son.el'la. L. fem. dim. suff. *-ella* diminutive ending; N.L. fem. n. *Pearsonella* named in honour of the British scientist Bruce Pearson, known for his contributions to the study of *Campylobacter*).

A bacterial genus identified by metagenomic analyses of human faeces. The genus includes all bacteria with genomes that show ≥60% average aminoacid identity to the genome of the type strain from the type species, *Candidatus* Pearsonella faecalis. This is a new name for the GTDB alphanumeric genus UBA1822. This genus has been assigned by GTDB-Tk v1.5.0 working on GTDB R06-RS202 reference data[1, 2] to the order *Veillonellales* and to the family *Dialisteraceae*

Description of *Candidatus* Pearsonella faecalis sp. nov.
Candidatus Pearsonella faecalis (fae.ca'lis. N.L. fem. adj. *faecalis*, of faeces).

A bacterial species identified by metagenomic analyses. This species includes all bacteria with genomes that show ≥95% average nucleotide identity to the type

genome for the species to which we have assigned the genome identifier hylon_MAXBIN__006 and which is available via NCBI BioSample SAMN18871199. This is a new name for the alphanumeric GTDB species sp002314995. The GC content of the type genome is 56.55 % and the genome length is 1.81 Mbp.

Description of *Candidatus* Physcomorpha gen. nov.
Candidatus Physcomorpha (Phys.co.mor'pha. Gr. fem. n. *physke* large intestine; Gr. fem. n. *morphe* a form, shape; N.L. fem. n. *Physcomorpha* a microbe associated with the large intestine).

A bacterial genus identified by metagenomic analyses of human faeces. The genus includes all bacteria with genomes that show ≥60% average aminoacid identity to the genome of the type strain from the type species, *Candidatus* Physcomorpha faecium. This is a new name for the GTDB alphanumeric genus UBA11524. This genus has been assigned by GTDB-Tk v1.5.0 working on GTDB R06-RS202 reference data [1, 2]to the order *Christensenellales* and to the family *CAG-74*

Description of *Candidatus* Physcomorpha faecium sp. nov.
Candidatus Physcomorpha faecium (fae'ci.um. L. fem. gen. pl. n. *faecium*, of faeces).

A bacterial species identified by metagenomic analyses. This species includes all bacteria with genomes that show ≥95% average nucleotide identity to the type genome for the species to which we have assigned the genome identifier rmaize_MAXBIN__031 and which is available via NCBI BioSample SAMN18871268. This is a new name for the alphanumeric GTDB species sp000437595. The GC content of the type genome is 57.78 % and the genome length is 3.22 Mbp.

Description of *Candidatus* Physconaster gen. nov.
Candidatus Physconaster (Phys.co.nas'ter. Gr. fem. n. *physke* large intestine; Gr. masc. n. *naster* an inhabitant N.L. masc. n. *Physconaster* a microbe inhabiting the large intestine).

A bacterial genus identified by metagenomic analyses of human faeces. The genus includes all bacteria with genomes that show ≥60% average aminoacid identity to the genome of the type strain from the type species, *Candidatus* Physconaster merdicola. This is a new name for the GTDB alphanumeric genus UBA11774. This genus has been assigned by GTDB-Tk v1.5.0 working on GTDB R06-RS202 reference data[1, 2] to the order *Lachnospirales* and to the family *Lachnospiraceae*

Description of *Candidatus* Physconaster merdicola sp. nov.
Candidatus Physconaster merdicola (mer.di'co.la. L. gen. fem. n. *merda*, faeces; L. masc./fem. suff. *-cola*, inhabitant of; N.L. fem. n. *merdicola* inhanbitant of faeces).

A bacterial species identified by metagenomic analyses. This species includes all bacteria with genomes that show ≥95% average nucleotide identity to the type genome for the species to which we have assigned the genome identifier T0_METABAT__254 and which is available via NCBI BioSample SAMN18871326. This is a new name for the alphanumeric GTDB species sp003507655. The GC content of the type genome is 41.91 % and the genome length is 2.16 Mbp.

Description of *Candidatus* Ruminococcus anthropi sp. nov.
Candidatus Ruminococcus anthropi (an.thro'pi. Gr. masc. n. *anthropos*, a human being; N.L. gen. masc. n. *anthropi*, of a human being).

A bacterial species identified by metagenomic analyses. This species includes all bacteria with genomes that show ≥95% average nucleotide identity to the type genome for the species to which we have assigned the genome identifier hylon_METABAT__215 and which is available via NCBI BioSample SAMN18871209. This is a new name for the alphanumeric GTDB species sp900314705. GTDB has assigned this species to genus with an alphabetic suffix which cannot be incorporated into a well-formed binomial, so in naming this species, we have used the basonym for the genus. The GC content of the type genome is 33.46 % and the genome length is 1.46 Mbp.

Description of *Candidatus* Ruminococcus hominis sp. nov.
Candidatus Ruminococcus hominis (ho'mi.nis. L. gen. masc. n. *hominis*, of a human being).

A bacterial species identified by metagenomic analyses. This species includes all bacteria with genomes that show ≥95% average nucleotide identity to the type genome for the species to which we have assigned the genome identifier rmaize_MAXBIN__013 and which is available via NCBI BioSample SAMN18871266. This is a new name for the alphanumeric GTDB species sp000433635. GTDB has assigned this species to genus with an alphabetic suffix which cannot be incorporated into a well-formed binomial, so in naming this species, we have used the basonym for the genus. The GC content of the type genome is 45.98 % and the genome length is 2.41 Mbp.

Description of *Candidatus* Sangerella gen. nov.
Candidatus Sangerella (San.ger.el'la. L. fem. dim. suff. *-ella* diminutive ending; N.L. fem. n. *Sangerella* named in honour of Frederick Sanger (1918-2013), British scientist; awarded the 1958 Nobel Prize in Chemistry for his work on the structure of protein and the 1980 Nobel Prize in Chemistry for inventing dideoxy sequencing).

A bacterial genus identified by metagenomic analyses of human faeces. The genus includes all bacteria with genomes that show ≥60% average aminoacid identity to the genome of the type strain from the type species, *Candidatus* Sangerella faecicola. This is a new name for the GTDB alphanumeric genus UBA737. This genus has been assigned by GTDB-Tk v1.5.0 working on GTDB R06-RS202 reference data [1, 2]to the order *Oscillospirales* and to the family *Acutalibacteraceae*

Description of *Candidatus* Sangerella faecicola sp. nov.
Candidatus Sangerella faecicola (fae.ci'co.la. L. fem. n. *faex, faecis* faeces; L. suff. -*cola* inhabitant of; N.L. fem. n. *faecicola* a microbe inhabitating faeces).

A bacterial species identified by metagenomic analyses. This species includes all bacteria with genomes that show ≥95% average nucleotide identity to the type genome for the species to which we have assigned the genome identifiers rmaize_MAXBIN__077 and T0_METABAT__221 and which is available via NCBI BioSample SAMN18871271. This is a new name for the alphanumeric GTDB species sp900549055. The GC content of the type genome are 47.36 % and 46.01 % and the genome lengths are 2.02 Mbp and 2.89 Mbp.

Description of *Candidatus* Splanchousia gen. nov.
Candidatus Splanchousia (Splanch.ou'si.a. Gr. neut. n. *splanchnon* guts; L. fem. n. *ousia* an essence; *Splanchousia* a microbe associated with the intestines).

A bacterial genus identified by metagenomic analyses of human faeces. The genus includes all bacteria with genomes that show ≥60% average aminoacid identity to the genome of the type strain from the type species, *Candidatus* Splanchousia colicola. This is a new name for the GTDB alphanumeric genus UBA1191. This genus has been assigned by GTDB-Tk v1.5.0 working on GTDB R06-RS202 reference data[1, 2] to the order *Peptostreptococcales* and to the family *Anaerovoracaceae*

Description of *Candidatus* Splanchousia colicola sp. nov.
Candidatus Splanchousia colicola (co.li'co.la. L. neut. n. *colum*, colon; L. masc./fem. suff. *-cola*, inhabitant of; N.L. fem. n. *colicola* inhabitant of the colon).

A bacterial species identified by metagenomic analyses. This species includes all bacteria with genomes that show ≥95% average nucleotide identity to the type genome for the species to which we have assigned the genome identifier avicell_METABAT__39 and which is available via NCBI BioSample SAMN18871194. This is a new name for the alphanumeric GTDB species sp900066305. The GC content of the type genome is 49.21 % and the genome length is 2.02 Mbp.

Description of *Candidatus* Splanchousia faecium sp. nov.
Candidatus Splanchousia faecium (fae'ci.um. L. fem. gen. pl. n. *faecium*, of faeces).

A bacterial species identified by metagenomic analyses. This species includes all bacteria with genomes that show ≥95% average nucleotide identity to the type genome for the species to which we have assigned the genome identifier rmaize_METABAT__166 and which is available via NCBI BioSample SAMN18871279. This is a new name for the alphanumeric GTDB species sp900549125. The GC content of the type genome is 47.53 % and the genome length is 2.11 Mbp.

Description of *Candidatus* Wallaceimonas gen. nov.
Candidatus Wallaceimonas (Wal.lace.i.mo'nas. L. fem. n. *monas* a monad; N.L. fem. n. *Wallaceimonas* named in honour of British naturalist Alfred Russel Wallace (1823-1913), co-discoverer of evolution by natural selection).

A bacterial genus identified by metagenomic analyses of human faeces. The genus includes all bacteria with genomes that show ≥60% average aminoacid identity to the genome of the type strain from the type species, *Candidatus* Wallaceimonas faecalis. This is a new name for the GTDB alphanumeric genus UMGS1696. This genus has been assigned by GTDB-Tk v1.5.0 working on GTDB R06-RS202 reference data[1, 2] to the order *Oscillospirales* and to the family *CAG-272*

Description of *Candidatus* Wallaceimonas faecalis sp. nov.
Candidatus Wallaceimonas faecalis (fae.ca'lis. N.L. fem. adj. *faecalis*, of faeces).

A bacterial species identified by metagenomic analyses. This species includes all bacteria with genomes that show ≥95% average nucleotide identity to the type genome for the species to which we have assigned the genome identifier T0_METABAT__60 and which is available via NCBI BioSample SAMN18871330. This is a new name for the alphanumeric GTDB species sp900753285. The GC content of the type genome is 49.14 % and the genome length is 1.81 Mbp.

Supplementary References

1. Chaumeil P-A, Mussig AJ, Hugenholtz P, Parks DH: **GTDB-Tk: a toolkit to classify genomes with the Genome Taxonomy Database.** *Bioinformatics* 2019, **36:**1925-1927.
2. Parks DH, Chuvochina M, Chaumeil P-A, Rinke C, Mussig AJ, Hugenholtz P: **A complete domain-to-species taxonomy for Bacteria and Archaea.** *Nat. Biotechnol.* 2020, **38:**1079-1086.

Supplementary Note 2 Media preparation materials, sources, and quantity[a]

Solution	Source	Quantity/litre
Fatty Acid Solution		
NaOH (0.2M)	Sigma-Aldrich, catalogue no 06203	1 L
Acetic acid	Sigma-Aldrich, catalogue no A6283	6.85 mL
Propionic acid	Sigma-Aldrich, catalogue no 402907	3.00 mL
Butyric acid	Sigma-Aldrich, catalogue no B103500	1.84 mL
Isobutyric acid	Sigma-Aldrich, catalogue no 58360	0.47 mL
2-Methylbutyric acid	Sigma-Aldrich, catalogue no W269514	0.55 mL
Valeric Acid	Sigma-Aldrich, catalogue no 240370	0.55 mL
Isovaleric acid	Sigma-Aldrich, catalogue no 129542	0.55 mL
Haemin Solution		
NaOH (0.05M)	Sigma-Aldrich, catalogue no 06203	1 L
Haemin		100.0 mg
Trace Mineral Solution		
HCl (0.02M)	Fisher Scientific, catalogue no 15676840	1 L
Manganese chloride ($MnCl_2$•$4H_2O$)	Sigma-Aldrich, catalogue no M3634	25.0 mg
Ferrous Sulphate ($FeSO_4$•$7H_2O$)	Sigma-Aldrich, catalogue no F7002	20.0 mg
Zinc chloride ($ZnCl_2$)	Sigma-Aldrich, catalogue no 793523	25.0 mg
Copper chloride ($CuCl$•$2H_2O$)	Sigma-Aldrich, catalogue no 307483	25.0 mg
Cobalt chloride ($CoCl_2$•$6H_2O$)	Sigma-Aldrich, catalogue no 255599	50.0 mg
Selenium dioxide (SeO_2)	Sigma-Aldrich, catalogue no 213365	50.0 mg
Nickel chloride ($NiCl_2$•$6H_2O$)	Sigma-Aldrich, catalogue no 223387	250.0 mg
Sodium molybdate (Na_2MoO_4•$2H_2O$)	Sigma-Aldrich, catalogue no 331058	250.0 mg
Sodium metavanadate ($NaVO_3$)	Sigma-Aldrich, catalogue no 590088	31.4 mg
Boric acid (H_3BO_3)	Sigma-Aldrich, catalogue no 31146	250.0 mg
Vitamin-Phosphate Solution (filtered sterilized using 0.2µm nylon filter)		
Potassium phosphate monobasic KH_2PO_4	Sigma-Aldrich, catalogue no P9791	20.4 mg
Biotin	Sigma-Aldrich, catalogue no B4639	20.6 mg
Folic acid	Sigma-Aldrich, catalogue no F8758	164.0 mg
Calcium D-pantothenate	Sigma-Aldrich, catalogue no P5155	164.0 mg
Nicotinamide	Sigma-Aldrich, catalogue no 72340	164.0 mg
Riboflavin	Sigma-Aldrich, catalogue no R9504	164.0 mg
Thiamine HCl	Sigma-Aldrich, catalogue no T4625	164.0 mg
Pyridoxine HCl	Sigma-Aldrich, catalogue no 181986	164.0 mg
Para-amino benzoic acid	Sigma-Aldrich, catalogue no A9878	20.4 mg
Cyanocobalamin (Vitamin B12)	Sigma-Aldrich, catalogue no C3607	20.6 mg
Reducing Agent (filtered sterilized using 0.2µm nylon filter)		
Deionized water at 100°C*		1L
L-Cysteine HCl	VWR, catalogue no ACRO434850010	20.0 g
Na_2S•$9H_2O$	Sigma-Aldrich, catalogue no 431648	20.0 g
Sodium Carbonate Solution (Na_2CO_3, degassed with CO2 and autoclaved) Sigma 223484		82g/L water*
Vitamin Phosphate+ Na_2CO_3 Solution		**75 mL total**
Na_2CO_3		60 mL
Vitamin Phosphate		15 mL
Resazurin Solution	Sigma-Aldrich, catalogue no R7017	1.0 g/L water*
Potassium Hydroxide (3M)	Sigma-Aldrich, catalogue no 221473	160 g/L water*
Basal Solution (degassed with CO_2 and autoclaved)		
Deionized water at 100°C*		1L
KCl	Sigma-Aldrich, catalogue no P3911	713.4 mg
NaCl	Sigma-Aldrich, catalogue no S7653	713.4 mg
$CaCl_2$•$2H_2O$	Sigma-Aldrich, catalogue no 223506	237.8 mg

$MgSO_4 \cdot 7H_2O$	Sigma-Aldrich, catalogue no	594.5 mg
Pipes buffer	Sigma-Aldrich, catalogue no P6757	1,783.5 mg
NH_4Cl	Sigma-Aldrich, catalogue no A9434	642.0 mg
Trypticase Peptone	VWR, catalogue no 1.07213.1000	1,189.0 mg
**Reagents**		
Resazurin solution		1.17 mL
Trace Mineral solution		11.89 mL
Haemin solution		11.89 mL
Fatty Acid solution		11.89 mL
KOH		Adjust pH 6.8

Supplementary *Table 1: Assembly statistics in base-pair, bp, unless otherwise stated*

Treatment	Number of contigs	N50 (Kbp)	Largest Contig	Total assembly length
**short read assembly (using Megahit)**				
**Avicell**	362,193	5.9	522,610	611,883,067
**Hylon**	359,933	4.9	807,467	566,999,279
**Inulin**	325,487	4.5	667,421	478,846,585
**Potato**	331,720	5.7	580,261	539,511,443
**R.maize**	317,785	4.7	703,349	487,894,115
**N.maize**	304,686	5.2	522,608	494,497,388
**Time 0h**	369,675	6.2	551,334	646,904,987
**Hybrid assembly (using OPERA-MS with Megahit and minimap2)**				
**Avicell**	336,341	10.0	1,204,096	619,505,714
**Hylon**	268,928	29.2	1,635,802	615,331,172
**Inulin**	236,720	36.0	1,544,219	532,364,427
**Potato**	303,698	10.8	1,632,261	550,284,682
**R.maize**	226,040	38.1	1,148,593	540,634,515
**N.maize**	288,406	7.7	1,052,519	499,371,327
**Time 0h**	278,441	33.5	1,381,878	694,064,798

Supplementary Table 2: GTDb taxonomy from the dereplication MAG clusters

Phylum	Family	Dereplicated genomes	No. of genus	No. of species	No. of MAGs
Actinobacteriota	*Bifidobacteriaceae*	5	1	5	29
	Coriobacteriaceae	1	1	1	7
	Eggerthellaceae	10	3	8	16
Bacteroidota	*Bacteroidaceae*	7	4	6	28
	Barnesiellaceae	1	1	1	7
	Marinifilaceae	3	2	2	6
	Rikenellaceae	7	3	6	21
	Tannerellaceae	1	1	1	6
	UBA11471	2	1	1	4
Desulfobacterota_A	*Desulfovibrionaceae*	1	1	1	7
Firmicutes	*CAG-1000*	1	1	1	1
	Erysipelotrichaceae	2	2	2	13
	Streptococcaceae	3	1	1	5
	UBA660	1	1	1	1
Firmicutes_A	*Acutalibacteraceae*	8	7	7	31
	Anaerotignaceae	1	1	1	1
	Anaerovoracaceae	4	2	2	7
	Butyricicoccaceae	8	2	2	12
	CAG-138	1	1	1	1
	CAG-272	1	1	1	1
	CAG-508	1	1	1	1
	CAG-74	3	2	2	15
	Lachnospiraceae	41	30	35	130
	Oscillospiraceae	22	7	9	79
	Ruminococcaceae	11	7	9	50
Firmicutes_C	*Dialisteraceae*	2	2	2	8
Proteobacteria	*Burkholderiaceae*	2	2	2	13
	Enterobacteriaceae	1	1	1	4
Verrucomicrobiota	*Akkermansiaceae*	1	1	1	7

Supplementary table 3: Genomes depicted as early and late degraders according to the time the genomes showed a 2x fold change.

Treatment	Early degraders		Late degraders	
	Cluster	Taxa (ordered by cluster)	Cluster	Taxa (ordered by cluster)
Avicell	cluster_111_0, cluster_26_1, cluster_28_0	*Blautia hydrogenotrophica, Escherichia coli, Candidatus* Splanchousia colicola	cluster_51_1, cluster_58_1, cluster_63_1	*Candidatus Caccadaptatus darwinii, Candidatus Minthonaster hominis, Faecalibacterium prausnitzii*
Hylon	cluster_104_1, cluster_26_1, cluster_32_1, cluster_41_0, cluster_84_1 cluster_65_1, cluster_72_1	*Pararoseburia caccae , Escherichia coli, Bifidobacterium adolescentis, Candidatus* Ruminococcus anthropi,*Ruminococcus bromii, Gemmiger qucibialis, Candidatus* Eisenbergiella faecalis	cluster_49_1, cluster_51_1, cluster_52_1, cluster_58_1, cluster_96_1	*Dysosmobacter segnis, Candidatus Caccadaptatus darwinii, Candidatus Enteromorpha quadrami, Candidatus Minthonaster hominis, Candidatus Blautia hennigii*
Inulin	cluster_29_1, cluster_38_1, cluster_82_1	*Candidatus* Colinsella sterocoris, *Holdemanella porci, Candidatus* Minthovivens enterohominis	cluster_18_1 cluster_4_1 cluster_49_2 cluster_51_1 cluster_56_1 cluster_58_1 cluster_63_1	*Bacteroides uniformis, Alistipes indistinctus, Dysosmobacter segnis, Candidatus* Caccadaptatus darwinii, *Candidatus* Minthonaster faecium, *Candidatus* Minthonaster hominis*, Faecalibacterium prausnitzii*
Potato	cluster_2_1 cluster_29_1 cluster_44_1 cluster_63_1 cluster_66_1 cluster_84_1 cluster_96_1	*Sutterella wadsworthensis, Candidatus* Colinsella sterocoris*, Candidatus Aphodonaster merdae, Faecalibacterium prausnitzii, Candidatus* Gemmiger merdicola, *Ruminococcus bromii, Candidatus* Blautia hennigii	cluster_8_1 cluster_48_1 cluster_51_1 cluster_62_1	*Alistipes shahii, Dysosmobacter welbionis, Candidatus* Caccadaptatus darwinii, *Candidatus* Colihabitans norwichensis
R.maize	cluster_17_1 cluster_19_1 cluster_2_1 cluster_27_1 cluster_4_1 cluster_43_1 cluster_48_1 cluster_51_1 cluster_58_1 cluster_87_0	*Bacteroides fragilis, Parabacteroides* diastonis, *Sutterella wadsworthensis, Candidatus* Splanchousia faecium, *Alistipes indistinctus, Bilophila wadsworthia, Dysosmobacter welbionis, Candidatus* Caccadaptatus darwinii, *Candidatus* Minthonaster hominis, *Candidatus* Minthomorpha faecalis	cluster_26_1 cluster_29_1 cluster_30_1 cluster_31_1 cluster_32_1 cluster_34_1 cluster_38_1 cluster_46_0	*Escherichia coli, Candidatus* Colinsella sterocoris, *Bifidobacterium animalis, Bifidobacterium catenulatum, Bifidobacterium adolescentis, Bifidobactrium longum, Holdemanella porci , Ruthenibacterium lactatiformans*
