# Supplementary material for: Hybrid metagenome assemblies link carbohydrate structure with function in the human gut microbiome
Source: Commun Biol. 2022 Sep 8;5:932. doi: 10.1038/s42003-022-03865-0 (PMC9458734; doi:10.1038/s42003-022-03865-0)
Supplement: Supplementary file 3 — Description of Additional Supplementary Files [file 42003_2022_3865_MOESM3_ESM.pdf]

Description of Additional Supplementary Files

File name: Supplementary Data 1
Description: Long read PromethION and short read Illumina read summary metrics.

File name: Supplementary Data 2
Description: MetaPhlAn3 Taxonomic profiles for all treatments.

File name: Supplementary Data 3
Description: Metabolic gene pathways normalised and stratified to copies per million (CPM) using HuMAnN3 tool

File name: Supplementary Data 4
Description: Fold-changes calculated between the copies per million counts of Time 6h, 12h and 24h with Time 0 for Normal maize

File name: Supplementary Data 5
Description: Fold-changes calculated between the copies per million counts of Time 6h, 12h and 24h with Time 0 for Hylon

File name: Supplementary Data 6
Description: Fold-changes calculated between the copies per million counts of Time 6h, 12h and 24h with Time 0 for Avicell

File name: Supplementary Data 7
Description: Fold-changes calculated between the copies per million counts of Time 6h, 12h and 24h with Time 0 for Inulin

File name: Supplementary Data 8
Description: Fold-changes calculated between the copies per million counts of Time 6h, 12h and 24h with Time 0 for Potato

File name: Supplementary Data 9
Description: Fold-changes calculated between the copies per million counts of Time 6h, 12h and 24h with Time 0 for Retrograded maize

File name: Supplementary Data 10
Description: Fold-changes with at least 0.5-fold (log2) shift in abundance relative to baseline (time 0h) for Avicell.

File name: Supplementary Data 11
Description: Fold-changes with at least 0.5-fold (log2) shift in abundance relative to baseline (time 0h) for Inulin.

File name: Supplementary Data 12
Description: Fold-changes with at least 0.5-fold (log2) shift in abundance relative to baseline (time 0h) for normal maize.

File name: Supplementary Data 13
Description: Fold-changes with at least 0.5-fold (log2) shift in abundance relative to baseline (time 0h) for hylon.

File name: Supplementary Data 14
Description: Fold-changes with at least 0.5-fold (log2) shift in abundance relative to baseline (time 0h) for potato.

File name: Supplementary Data 15
Description: Fold-changes with at least 0.5-fold (log2) shift in abundance relative to baseline (time 0h) for retrograded maize.

File name: Supplementary Data 16
Description: MAG genomic statistics, assembly features, closest taxonomy annotation and relative evolutionary distance for genus and species not previously identified in NCBI.

File name: Supplementary Data 17
Description: MAGs with primary and secondary clusters

File name: Supplementary Data 18
Description: Latin binomials for MAGs without one. Latin taxa names for Genbank submission.

File name: Supplementary Data 19
Description: Comparison of genome statistics between MAGs from this study and GTDb corresponding representative MAG cluster.

File name: Supplementary Data 20
Description: Relative abundance, fold change and logratio foldchange for all MAGs.

File name: Supplementary Data 21
Description: CAZyme counts for all MAG clusters.

File name: Supplementary Data 22
Description: PULs identified in MAG clusters

File name: Supplementary Data 23
Description: CAZyme counts for all MAG clusters.

File name: Supplementary Data 24
Description: Abundance of CaZymes relative to the abundance of each MAG

File name: Supplementary Data 25
Description: Abundance of CaZymes that have a Signal peptide relative to the abundance of each MAG

File name: Supplementary Data 26
Description: Biosample accession numbers for bioproject submitted to SRA
