# Supplementary material for: Hybrid metagenome assemblies link carbohydrate structure with function in the human gut microbiome
Source: Commun Biol. 2022 Sep 8;5:932. doi: 10.1038/s42003-022-03865-0 (PMC9458734; doi:10.1038/s42003-022-03865-0)
Supplement: Supplementary file 5 — Reporting Summary [file 42003_2022_3865_MOESM5_ESM.pdf]

nature portfolio

Corresponding author(s): Frederick Warren

Last updated by author(s): Jun 27, 2022

Reporting Summary

Nature Portfolio wishes to improve the reproducibility of the work that we publish. This form provides structure for consistency and transparency in reporting. For further information on Nature Portfolio policies, see our Editorial Policies and the Editorial Policy Checklist.

Statistics

For all statistical analyses, confirm that the following items are present in the figure legend, table legend, main text, or Methods section.

n/a	Confirmed	
☐	☒	The exact sample size (*n*) for each experimental group/condition, given as a discrete number and unit of measurement
☐	☒	A statement on whether measurements were taken from distinct samples or whether the same sample was measured repeatedly
☐	☒	The statistical test(s) used AND whether they are one- or two-sided<br>*Only common tests should be described solely by name; describe more complex techniques in the Methods section.*
☒	☐	A description of all covariates tested
☒	☐	A description of any assumptions or corrections, such as tests of normality and adjustment for multiple comparisons
☐	☒	A full description of the statistical parameters including central tendency (e.g. means) or other basic estimates (e.g. regression coefficient) AND variation (e.g. standard deviation) or associated estimates of uncertainty (e.g. confidence intervals)
☐	☒	For null hypothesis testing, the test statistic (e.g. *F*, *t*, *r*) with confidence intervals, effect sizes, degrees of freedom and *P* value noted<br>*Give P values as exact values whenever suitable.*
☒	☐	For Bayesian analysis, information on the choice of priors and Markov chain Monte Carlo settings
☒	☐	For hierarchical and complex designs, identification of the appropriate level for tests and full reporting of outcomes
☒	☐	Estimates of effect sizes (e.g. Cohen's *d*, Pearson's *r*), indicating how they were calculated

Our web collection on statistics for biologists contains articles on many of the points above.

Software and code

Policy information about availability of computer code

Data collection	Guppy version 3.0.5+45c3543 – Basecalling in high accuracy mode
Data analysis	Linux-run tools:<br>Qcat version 1.1.0 – ONT demultiplexed<br>NanoStat version 1.1.2 – Sequence metrics ONT<br>Porechop version 0.2.3- ONT quality trimming and adapter removal<br>fastP version 0.20.0- Illumina quality trimming and adapter removal<br>MetaphlAn3 version 3.0.2 – Taxonomic profiling of illumina reads- Marker information dataset CHOCOphlAn 2019<br>HUMAnN version 3.0.2 - Functional gene annotation<br>Hclust2 1.0.0 - hierarchical clustering of taxonomic profiles<br>Megahit version 1.1.3- Contig assembly of Illumina reads<br>OPERA-MS version 0.8.2- hybrid assembly<br>Bowtie2 version 2.3.4.1 – Mapping reads to co-assemblies<br>Samtools version 1.10<br>MaxBin2 version 2.2.6 and MetaBat2 version 2.12.1 – Binning reads to MAGs<br>Anvi'o version 6.1 – Manual refinement and visual inspection of MAGs. Scripts used: Anvi-interactive, Anvi-run-hmms, Anvi-profile, Anvi-refine, Anvi-summarize<br>DAS tool version 1.1.2 – Aggregate MAGs into high-quality MAGs for each treatment<br>CheckM version 1.0.18 – assign completion and contamination scores for MAGs<br>dRep version 2.5.0 – produce dereplicated set of MAGs<br>GTDb version 0.3.5 with database R95- obtain closest taxonomic assignment for the MAGs

PhylophlAn version 0.99 – Phylogenetic tree
dbCAN version 2.0.11, CAZyDB (cazy database for dbCAN) version 2019, HMMdb (HMMER database for dbCAN) version 8

R-based tools:
RStudio version 1.1.453
ape version 5.3- Principle Coordinate Analyses
vegan package version 5.3
gtools version 3.5.0 – calculate foldchange and log ratios
ggtree version 2.2.4
ggplot2 version 3.3.2
dplyr version 1.0.2
aplot version 0.0.6
ComplexHeatmap version 2.4.3

Online tools
iTol version 4.3.1

Softwares
Inkscape version 1.0.1

For manuscripts utilizing custom algorithms or software that are central to the research but not yet described in published literature, software must be made available to editors and reviewers. We strongly encourage code deposition in a community repository (e.g. GitHub). See the Nature Portfolio guidelines for submitting code & software for further information.

Data

Policy information about availability of data

All manuscripts must include a data availability statement. This statement should provide the following information, where applicable:

- Accession codes, unique identifiers, or web links for publicly available datasets
- A description of any restrictions on data availability
- For clinical datasets or third party data, please ensure that the statement adheres to our policy

Raw read data from the PromethION and NovoSeq sequencing runs can be accessed through the NCBI SRA project number PRJNA722408 and can be accessed at http://www.ncbi.nlm.nih.gov/bioproject/722408. GenBank accession numbers for individual MAG's within this ProjectID can be found in Supplementary Table 5

Human research participants

Policy information about studies involving human research participants and Sex and Gender in Research.

Reporting on sex and gender	As a single sample was used for this proof-of-concept study, no information on sex or gender was included in this study
Population characteristics	Adult (≥ 18 years old), free-living, healthy donor who had not taken antibiotics in the 3 months prior to donation and was free from gastrointestinal disease
Recruitment	Recruitment was carried out through an anonymised database of study participants
Ethics oversight	Ethical approval was granted by the Human Research Governance Committee at the Quadram Institute (IFR01/2015) and the London - Westminster Research Ethics Committee (15/LO/2169).

Note that full information on the approval of the study protocol must also be provided in the manuscript.

Field-specific reporting

Please select the one below that is the best fit for your research. If you are not sure, read the appropriate sections before making your selection.

☒ Life sciences ☐ Behavioural & social sciences ☐ Ecological, evolutionary & environmental sciences

For a reference copy of the document with all sections, see nature.com/documents/nr-reporting-summary-flat.pdf

Life sciences study design

All studies must disclose on these points even when the disclosure is negative.

Sample size	This paper is primarily a proof-of-concept for novel sequencing technologies carried out in vitro so a single human stool was used, replicated across 6 independent in vitro model systems
Data exclusions	No data exclusions

Replication	As a proof-of-concept in vitro study technical replicates were not included
Randomization	Randomization was not relevant as this was an in vitro study
Blinding	Blinding was not relevant as this was an in vitro study

Reporting for specific materials, systems and methods

We require information from authors about some types of materials, experimental systems and methods used in many studies. Here, indicate whether each material, system or method listed is relevant to your study. If you are not sure if a list item applies to your research, read the appropriate section before selecting a response.

Materials & experimental systems

n/a	Involved in the study
☒	☐ Antibodies
☒	☐ Eukaryotic cell lines
☒	☐ Palaeontology and archaeology
☒	☐ Animals and other organisms
☒	☐ Clinical data
☒	☐ Dual use research of concern

Methods

n/a	Involved in the study
☒	☐ ChIP-seq
☒	☐ Flow cytometry
☒	☐ MRI-based neuroimaging
